# Exploring Emission Ratios: Influence of Neighboring Groups on TSAL Core

**DOI:** 10.1021/acs.jpca.5c01560

**Published:** 2025-03-27

**Authors:** Olaf Morawski, Pawel Gawrys, Marzena Banasiewicz, Cristina A. Barboza

**Affiliations:** Institute of Physics, Polish Academy of Sciences, Al. Lotników 32/46, 02-668 Warsaw, Poland

## Abstract

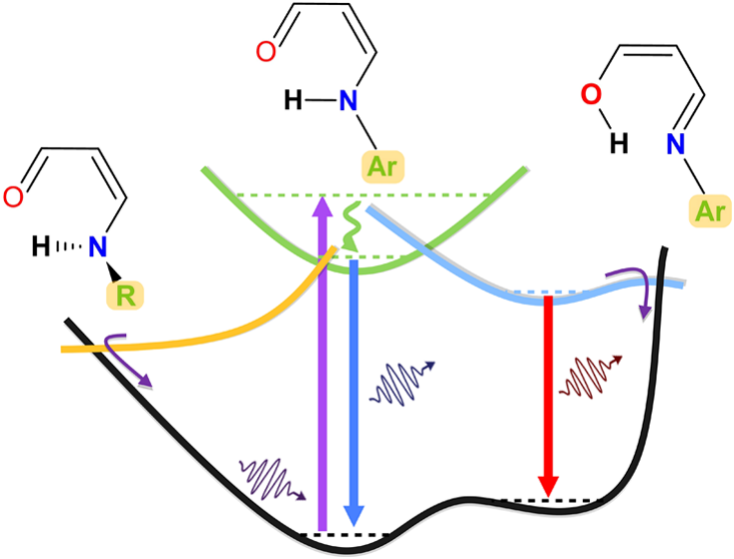

The photophysics of tris(salicylidenealdimines) (TSALs) has been examined, as they offer the possibility of populating multiple emissive species through the excited-state intramolecular proton transfer (ESIPT) reaction, resulting in broad visible spectrum coverage, a desirable property for white organic light-emitting diodes (wOLEDs). In this contribution, the synthesis and photophysical characterization of a new TSAL derivative, **C16**, are reported. It displays a distinct emission profile compared to previously studied tris(salicylideneanilines), with an inversion of the ESIPT/LE emission intensity ratio, exhibiting almost exclusively tautomer emission upon excitation in less polar solvents. *Ab initio* calculations suggest that the absence of emission from the locally excited (LE) state in nonpolar solvents may be related to the competition between two processes: ESIPT and *N*-pyramidalization of the enaminic nitrogen. Conversely, in polar aprotic solvents, the planar LE conformation is stabilized, and dual emission is observed. In protic solvents, however, the ESIPT reaction is suppressed due to competition with intermolecular hydrogen bonding interactions.

## Introduction

The excited-state intramolecular proton transfer (ESIPT) reaction often occurs in molecules that include heterocyclic nitrogen and/or oxygen atoms at moieties susceptible to proton transfer, resulting in multiple emission bands as a result of the population of different tautomeric species.^1−4^ The occurrence of ESIPT is strongly influenced by the solvent polarity and temperature, along with other factors that affect the emission profiles and the ratio between intensities of emission bands for the normal and tautomer species.^5−7^ ESIPT’s importance lies in its applications in organic light-emitting diodes,^8,9^ fluorescent probes,^10,11^ photochromic switches,^12,13^ sunscreen components,^14^ and chemosensors, among others.^15^

Tris(salicylidenealdimines) (TSALs) are a class of ESIPT-capable emitters having three proton transfer sites (three *keto*-enamine moieties), offering the potential to populate more than one emissive species, which can lead to broad coverage of the visible spectrum.^16^ Despite having three proton transfer sites, generally, aryl TSALs exhibit almost exclusively local emission upon excitation, regardless of the solvent used.^17,18^ Thus, it remains a challenge to populate tautomeric species from these types of molecules. The main deactivation channels for these compounds are related to large-amplitude movements around the iminic bridge, particularly the rotation of the C=N moiety in the tautomer species, as its energy would be significantly lower than that of the LE state, consequently decreasing the intensity of the band assigned to the tautomer emission. Polar solvents further stabilize this state due to its increased polarity. The interplay between ESIPT and LE bands is complex, influenced by functionalization through electronic^17^ and steric effects.^18^ The formation of an intermediate species in ESIPT-capable molecules with the proton transfer site pyramidalized, leads to an energy minimum with biradical character upon the irradiation of 2-(2′-hydroxyphenyl)benzimidazoles, resulting in the population of a dark twisted state,^19^ which may significantly increase the internal conversion rate of organic chromophores such as nitroaromatics and coumarins.^20,21^

**Scheme 1:**
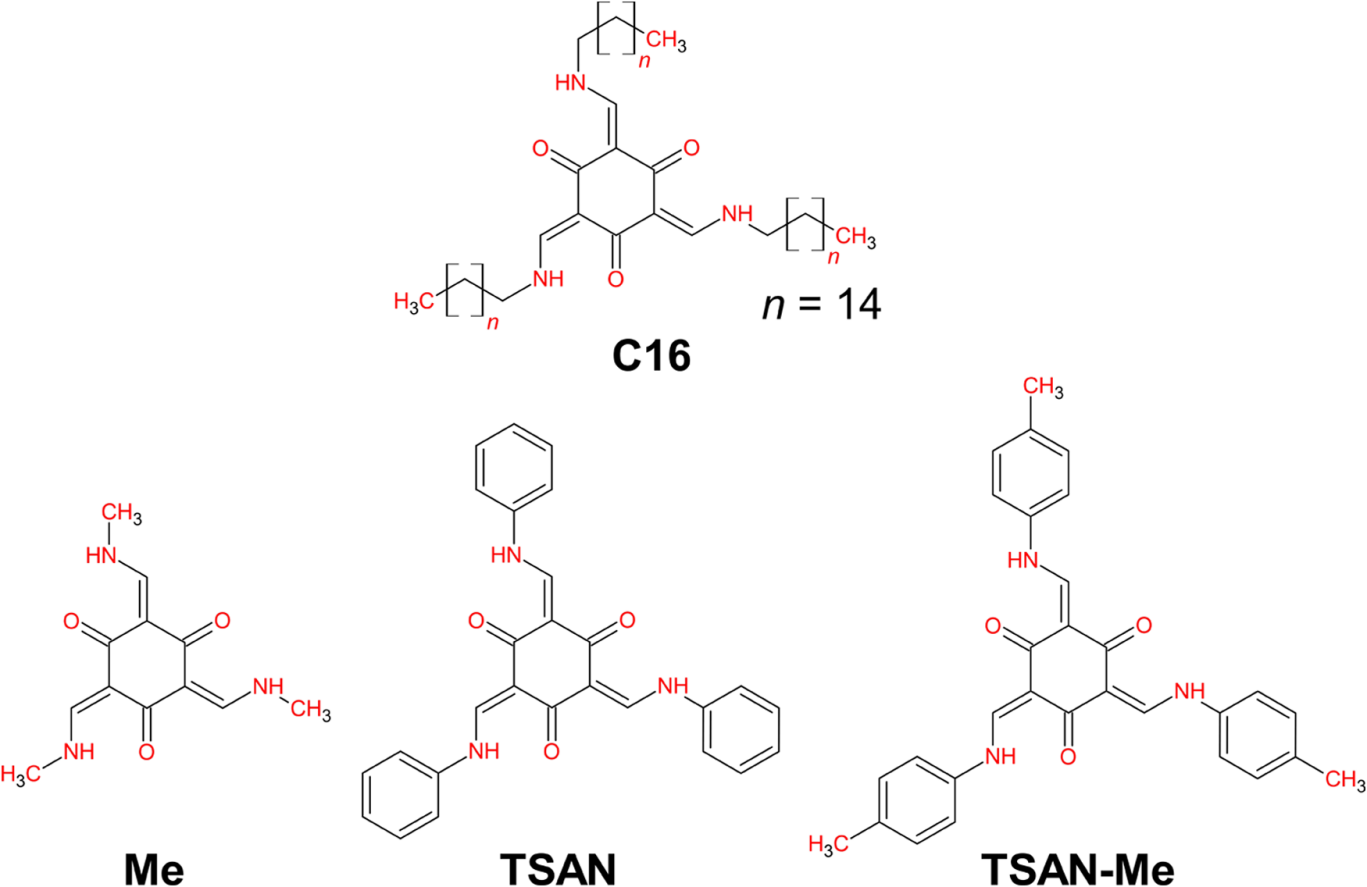
Molecular Structures of *cis-kkk* Diastereoisomers of **C16** and the Model Systems **Me**, **TSAN**, and **TSAN-Me**

The ESIPT process mechanism in organic chromophores has been the subject of extensive investigation, combining theoretical methods with spectroscopic techniques.^17,18,22,23^ However, standard density functional theory (DFT) calculations for molecules with significant long-range interactions, such as N–H···O hydrogen bonds present in TSALs, may lead to unreliable descriptions and inaccuracies in predicting reaction pathways.^24^ Therefore, in this contribution, we present theoretical explorations of the ESIPT mechanism for three model compounds: **TSAN**, **TSAN-Me**, and **Me**. Scheme 1 shows the most stable structures of these compounds, which have all labile hydrogens attached to nitrogen atoms at the proton transfer sites, designated as *kkk*, in which *k* corresponds to *keto* tautomer. Multiple TSAL rotamers may also be populated, with *cis* and *trans* species corresponding to the *C*_3*h*_ and *C*_*s*_ point groups, respectively. However, this symmetry applies only to the arrangement of peripheral groups attached to the C=C bond. Tris(salicylideneanilines) (TSANs) are a subclass of TSALs in which the iminic nitrogen is bonded to an aryl group. In contrast, **C16**, which has an aliphatic *N*-substituent (−C_16_H_33_), can be classified as a tris(salicylidenealkylamine). This distinction is important due to the unusual photophysics observed for the latter, which exhibits almost exclusively pure ESIPT emission, as disclosed in this article (*vide infra*). Consequently, *ab initio* calculations for **Me**, a model system for **C16** with truncated alkyl chains, were performed to investigate the mechanisms responsible for the inverted ESIPT/LE ratio in **C16** compared to those in related TSANs.

## Computational Details

The photoisomerization of the model system **Me** was evaluated at the *ab initio* level of theory using the Møller–Plesset perturbation theory (MP2)^25^ and second-order algebraic diagrammatic construction ADC(2).^26−28^ The correlation-consistent valence double-ζ basis set with polarization functions on all atoms (cc-pVDZ) was used.^29^ All calculations were performed using the TURBOMOLE 7.2 program package, applying the resolution of the identity (RI) approximation for the evaluation of the electron-repulsion integrals.^30,31^ The ground state equilibrium geometry (*S*_0_) was optimized using the MP2 method, while vertical excitation energies for the lowest six excited states, oscillator strengths, and the first singlet excited-state (*S*_1_) optimized geometries were computed within the ADC(2) approximation. Due to the ultrafast time scale of ESIPT reaction, its barrier was estimated by a relaxed scan along the N–H single bond constraining the molecule to the *C*_*s*_ symmetry.

## Results and Discussion

### Computational Analysis

This section is divided into two parts. At first, the evaluation of the structural and electronic properties of the **Me** derivative is presented. Then, the occurrence of photoreactions for it was explored in contrast to the parent model compounds previously published called **TSAN** and **TSAN-Me** shown in Scheme 1.^17,32^

#### Tris(salicylidenemethylamine)—**Me**

Geometry optimizations were performed for **Me** at the MP2/cc-pVDZ level of theory for its all-*enamine* conformation, having all labile hydrogens attached to nitrogen atoms in the proton transfer sites (*cis*-*kkk*) without symmetry constraints. The optimized structure is nearly planar. The possibility of the population of rotamers in the ground state was also considered by the twist of one dimethylamino group attached to the core, namely, *trans*-*kkk* (Scheme S1). Obtained results suggest that both isomers are almost degenerate (Δ*E* = 0.03 eV). Thus, it may be expected that both species would be thermally populated in the ground state, in agreement with the ^1^NMR measurements presented in the ESI.

Low-lying singlet states were computed at the ADC(2)/cc-pVDZ level of theory, as detailed in Table S1a. For the *cis*-*kkk* conformer, two bright degenerate states were found with energies of 4.21 eV (*f* = 0.55). This degeneracy arises from the near 3-fold symmetry of the structure, making these states susceptible to Jahn–Teller instability. In contrast, the *trans*-*kkk* rotamer exhibits lower symmetry and the lift of the bright state degeneracy, resulting in energies of 4.09 eV (*f* = 0.44) and 4.27 eV (*f* = 0.58) for its excited states. In consequence, *trans-kkk* is more polar than its all-symmetric counterpart. Computed transitions for both isomers can be assigned as locally excited (LE) vertical states without significant contributions from molecular orbitals (Table S1b) of methyl groups in the core periphery and low total dipole moments. However, due to the small difference in the bright states excitation energies between the isomers (less than 0.1 eV, lower than the error associated with ADC(2) calculations), it is unlikely to distinguish their electronic properties. Thus, further calculations were performed only for the most stable *cis*-*kkk* isomer. Henceforth, “*cis-kkk*” will be referred to as “*kkk.*”

**Scheme 2:**
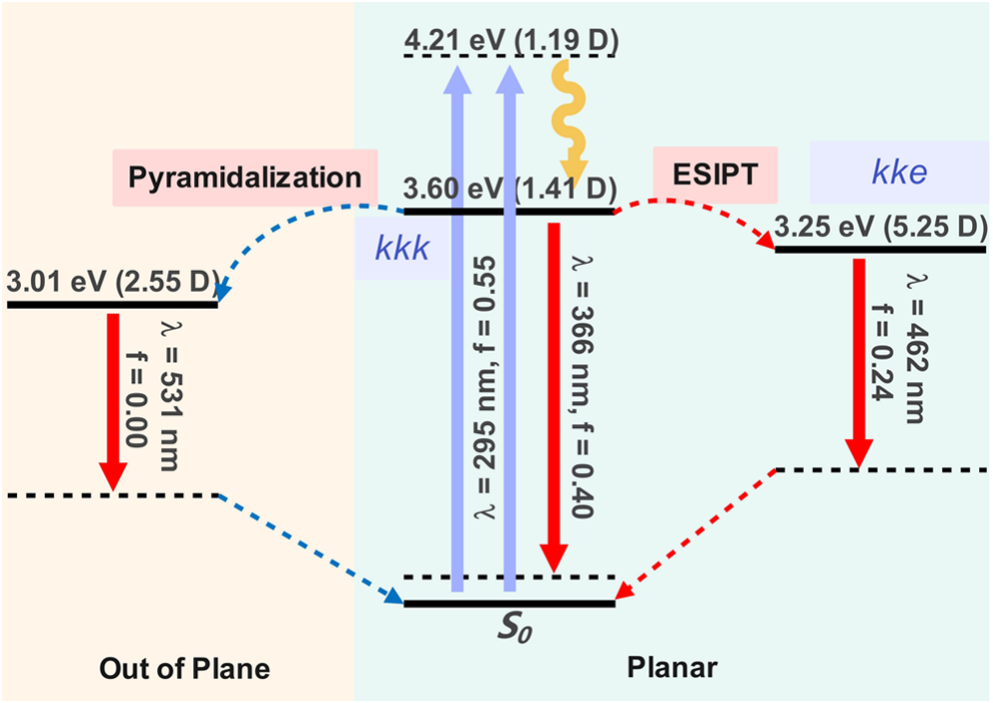
General Photophysical Scheme for **Me** Computed at the ADC(2)/cc-pVDZ Level of Theory: Absorption (Blue), Fluorescence (Red), and Internal Conversion (Yellow) Solid and dashed horizontal bars represent the adiabatic and vertical energies, respectively, of the absorbing/fluorescing state calculated at the optimized geometry of the respective states. Barriers for photoreactions predicted to occur upon excitation to *S*_1_ are depicted as red and blue dashed arrows for the ESIPT (*kkk* → *kke*) and enaminic nitrogen pyramidalization, respectively. Energies (eV) are given with respect to its ground state equilibrium structure and dipole moments are shown in round brackets (in Debye).

The optically active states computed for *kkk* are preceded by two nπ* dark states. The energy difference between the second (nπ*) and third (ππ*) vertically excited states is about 0.03 eV. The almost degeneracy between nπ* and ππ* energies of *kkk* is expected to result in the strong mixing of these states, which would allow *S*_1_ and *S*_2_ to gain some transition moment from the bright *S*_3_ and *S*_4_ states and contribute to optical absorption. Such close proximity of energies for low-lying nπ* and ππ* vertical excited states have not been observed for previous TSALs.^17,32^ The overlap between bright and dark pairs of vertical states (*O*_*ij*_) were estimated by
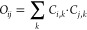
where *C*_*i*,*k*_ and *C*_*j*,*k*_ are the coefficients for configuration *k* in states *i* and *j*, respectively. The overlap values obtained for **Me** reveal significant mixing between dark nπ* states and bright ππ* states, particularly for pairs *S*_1_ – *S*_3_ and *S*_1_ – *S*_4_, estimated to be around 0.8 (Table S3). This mixing can be related to the Jahn–Teller distortion, which would further stabilize out of plane conformations, thus favoring the population of dark nπ* states (Scheme 2).

All calculations were performed in the gas phase. However, since the quenching of the ESIPT reaction was previously observed in the fluorescence of salicylaldimines in protic solvents,^33,34^ the microsolvation of **Me** with each proton transfer site surrounded by a methanol molecule suggests the formation of intermolecular hydrogen bonds (Figure S2), resulting in a nonemissive distorted structure with longer O···H bond distances. Such distortion effectively competes with the population of tautomers in the excited state, significantly decreasing the fluorescence quantum yield. Thus, in protic solvents, only the LE state of **Me** is predicted to be populated.

#### Emission Mechanisms of **Me** with Respect to **TSAN** and **TSAN-Me**

**Figure 1:**
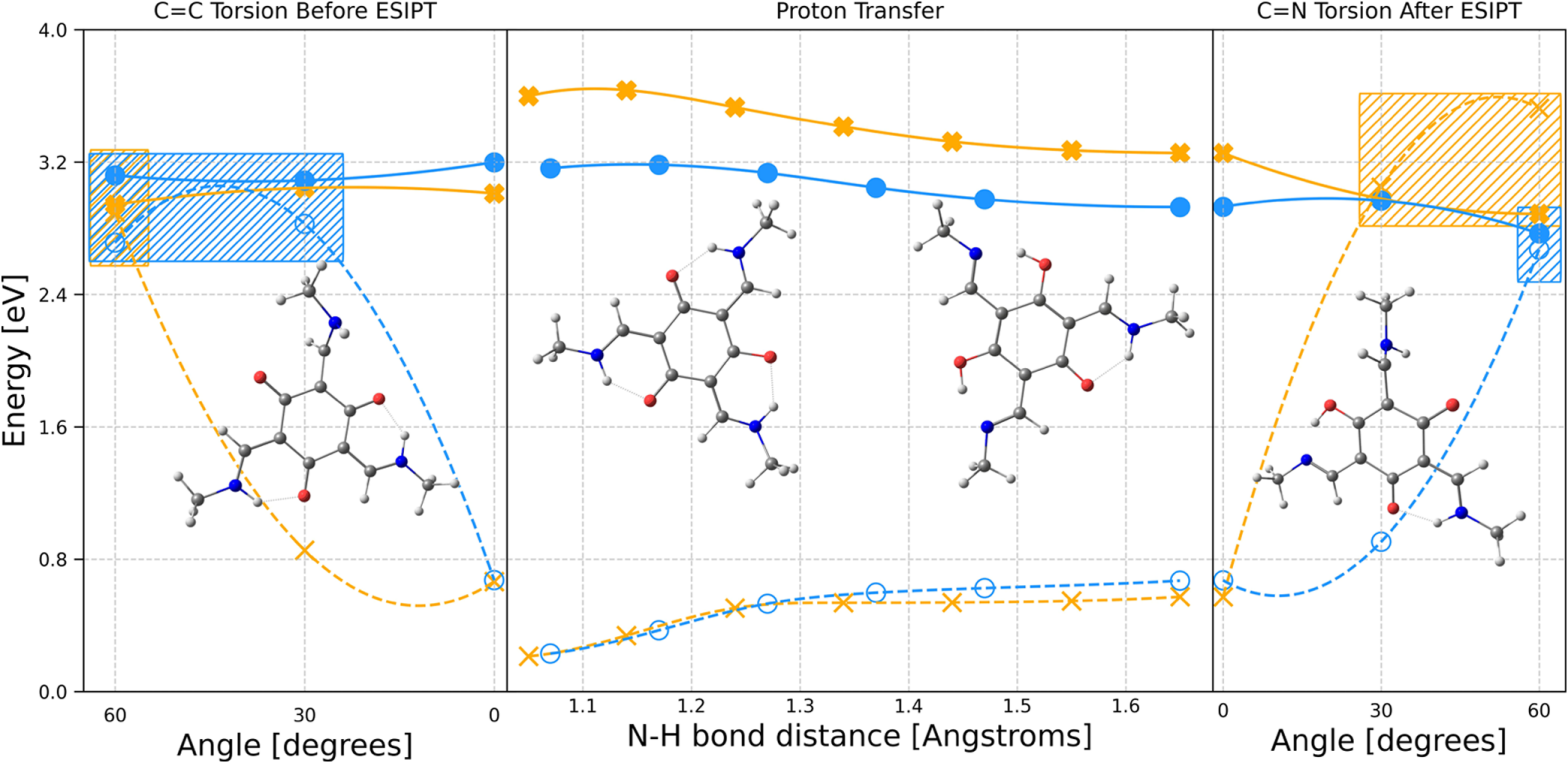
(Middle panel) Minimum energy profiles for the ESIPT (*kkk* → *kke*) reactions of **Me** (X orange markers) and **TSAN** (blue circles) predicted to occur upon excitation computed at the ADC(2)/cc-pVDZ level of theory to the first singlet excited state (*S*_1_), including their respective main nonradiative channels of deactivation and the twist along C=C (left panel) and C=N (right panel) dihedral angles, through which the *S*_1_/*S*_0_ conical intersection could be reached (hatched areas). The molecular structures presented in the middle panel correspond to the *kkk* and *kke* tautomers, while conformations shown in the left and right panels correspond to the molecular structures of *kkk* and *kke* for which the conical intersections are predicted to be reached before and after ESIPT, respectively.

Minimum energy paths were computed by using the ADC(2)/cc-pVDZ level of theory. Previous theoretical investigations explored the possibility of multiple emissions from several TSAL derivatives. In general, the ESIPT reaction (*kkk* → *kke*) is predicted to occur in this class of molecules in a barrierless fashion, populating a tautomer with increased polarizability, resulting in a pronounced reduction of the *S*_1_/*S*_0_ energy gap with respect to the parent compound *kkk*.^17,18,32^ It would also have seamless access to the conical intersection upon the occurrence of large-amplitude movements such as the twist along C=C and C=N double bonds, before and after ESIPT, respectively, as can be seen for TSAN in Figure 1 (blue). Despite the similarity of molecular structure, there is a discontinuity between the first and second panels for **Me** (X orange markers). This feature may be related to the population of a conformer, with the nitrogen pyramidalized proton transfer site that does not participate to the ESIPT reaction. The out of plane distortion would lead to a significantly more stable structure, with adiabatic energy about 0.6 eV lower than the planar LE minimum, competing with its fluorescence (Scheme 2). As a consequence, fluorescence from LE is expected to be quenched in nonpolar solvents, leading to a predominance of the tautomer *kke* emission. In contrast, in polar media, the planar LE conformation is favored, resulting in a lower ESIPT/LE emission ratio. In addition, the stabilization of the LE state increases the ESIPT reaction barrier and decreases the *kke* emission energy due to its higher dipole moment compared to that of *kkk*, which facilitates access to the conical intersection with the ground state. Consequently, in polar solvents, only local emission is expected, consistent with the decay times of the red fluorescence band reported in Table 1.

**Table 1:** Optical Data for Studied **C16**^a^

solvent	λ_abs max_ λ_fl max_ (nm)	Φ (%)	τ (ns)
*n*-hexadecane	353sh, **341**, 291, 247vib	2.9	1.36
	395vw (local), **536** (ESIPT)		
*n*-hexane	354sh, **341**, 290, 247vib	2.6	1.33
	395vw (local), **538** (ESIPT)		
toluene	**342**, 294	1.5	0.72
	392w (local), **536** (ESIPT)		
THF	**341**, 295	1.1	0.67
	395wsh (local), **533** (ESIPT)		
1-octOH	**337**, 299, 242	0.33	0.49
	395sh, 445sh, **512**		
MeOH	**334**, 300, 238	0.25	0.36
	**437**		
DMF	**341**, 295	0.48	0.38
	440sh, **535** (ESIPT)		

a The absorption/emission peaks of highest intensity are in bold. Legend: sh—shoulder, shw—weak shoulder, vib—vibronic structure, Φ—fluorescence quantum yield of the total spectrum, τ—decay time of the red band of fluorescence spectrum.

### Experimental Studies

The synthesis of **C16** is available in the ESI. **Me** was briefly characterized by optical spectroscopy, as well. However, **Me** was more difficult to prepare in the pure state, and its physical appearance was inconvenient for solid-state measurements. **C16** in view of its much more trivial synthesis and very good stability gives justification for the preference to use TSAL with long *N*-alkyl chain in this study. **Me** and **C16** exhibit identical absorption, emission, and fluorescence excitation spectra (Figures S17, S18, S19), demonstrating that the length of the alkyl chain does not affect the photophysics of TSALs.

#### Optical Spectra

**Figure 2:**
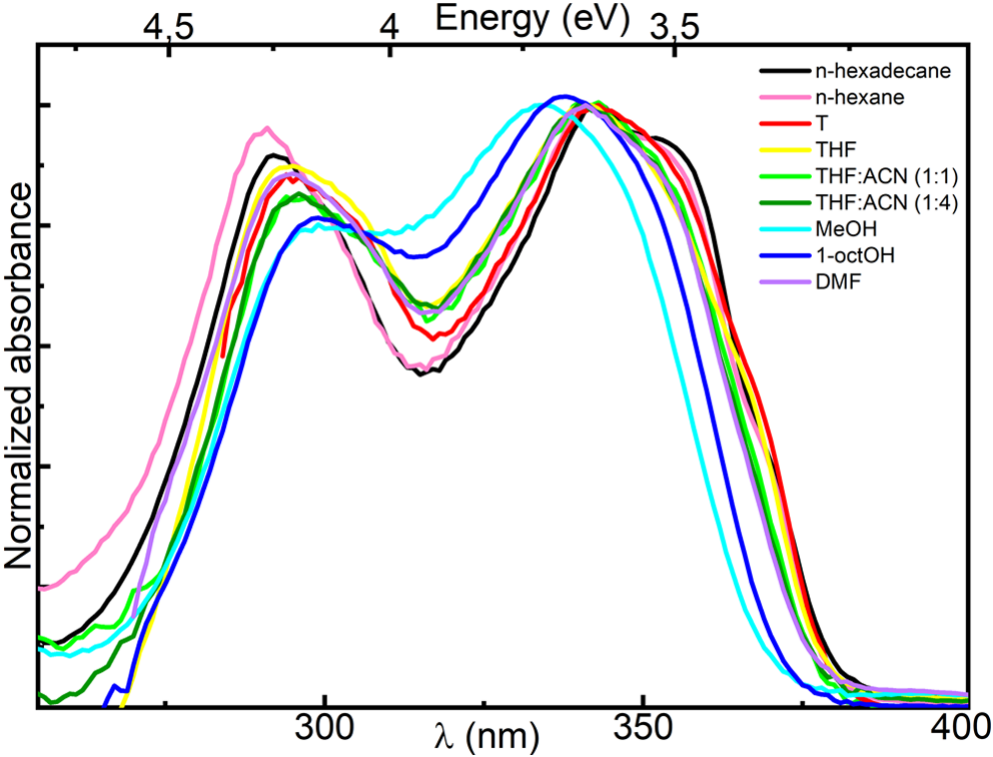
Normalized absorption spectrum of **C16** in solvents at room temperature.

Normalized absorption spectra of **C16** are depicted in Figure 2, and the relevant data on their band positions are listed in Table 1. The spectrum measured in *n*-hexane shows three bands at 291, 341, and 353 nm, wavelengths similar to those observed for other TSAL derivatives published elsewhere.^17,18^ Thus, replacing the aryl group of TSANs with an alkyl chain in the *keto*-enamine moiety of **C16** does not significantly affect the positions and relative intensities of absorption bands, in agreement to the predicted vertical excited states shown in Table S1a.

**Figure 3:**
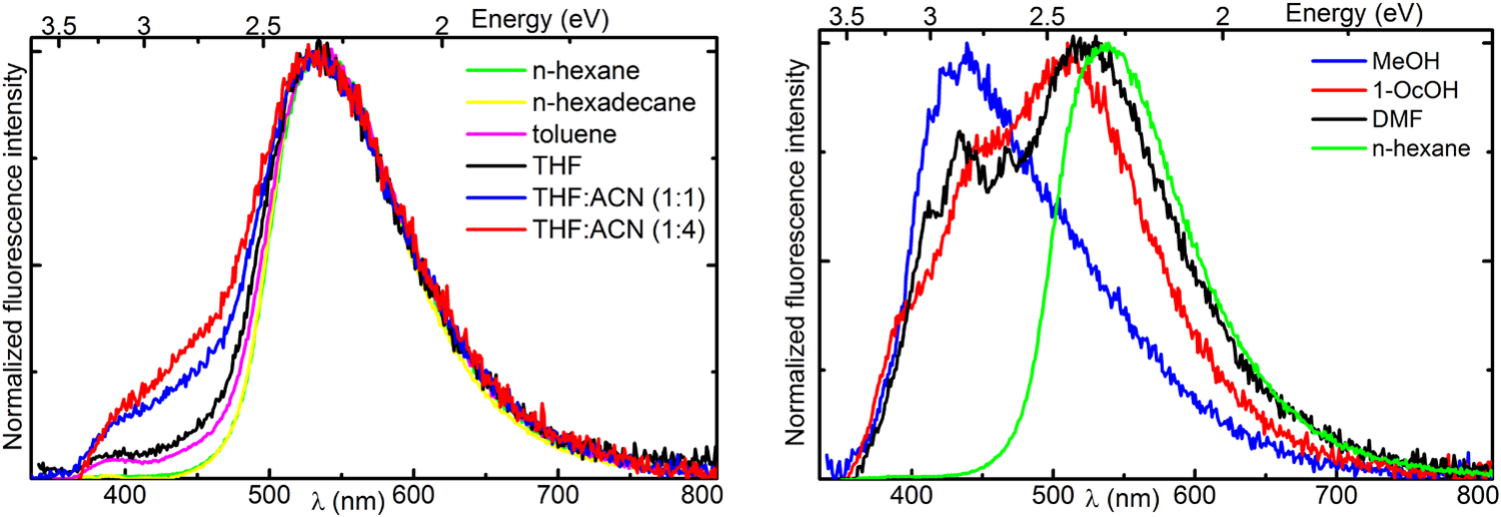
Normalized fluorescence spectrum of **C16** measured in (left) nonprotic and (right) protic solvents at room temperature. Excitation wavelength used was 340 nm.

The absorption spectra measured using tetrahydrofuran (THF), toluene, and *n*-hexadecane are similar, despite their distinguished properties (being slightly polar, polarizable, and viscous, respectively). A slight solvatochromism that can be observed only with the use of more polar solutions, such as mixtures of THF and acetonitrile (ACN), confirms negligible change of dipole moment between *S*_0_ and LE states, as predicted for the *kkk* structure (Table S1a). A similar trend was previously observed for TSANs^17,18^ and can be related to the high symmetry of the molecular core of **C16** resulting in low computed transition dipole moments. Also with dimethylformamide (DMF) (highly polar (ε = 36.7), nonprotic (hydrogen-bond donor acidity parameter, α = 0.0) and basic (hydrogen-bond acceptor, basicity β = 0.69)),^35^ the lowest energy band slightly shifts to shorter wavelengths, in contrast to polar and protic solvents. When the latter is used (1-octanol or methanol), a pronounced blue shift is observed (Figure 3), typical of states with nπ* character. This band displacement is more pronounced in methanol than in the less protic 1-octanol (α = 0.93 and 0.78, respectively).^35^ In addition, the hypsochromic shift observed in hydrogen-accepting DMF is significantly lower than in hydrogen-donating solvents (methanol and 1-octanol). These observations support theoretical predictions suggesting the ππ* – nπ* vertical excited states mixing given in Table S3.

**Figure 4:**
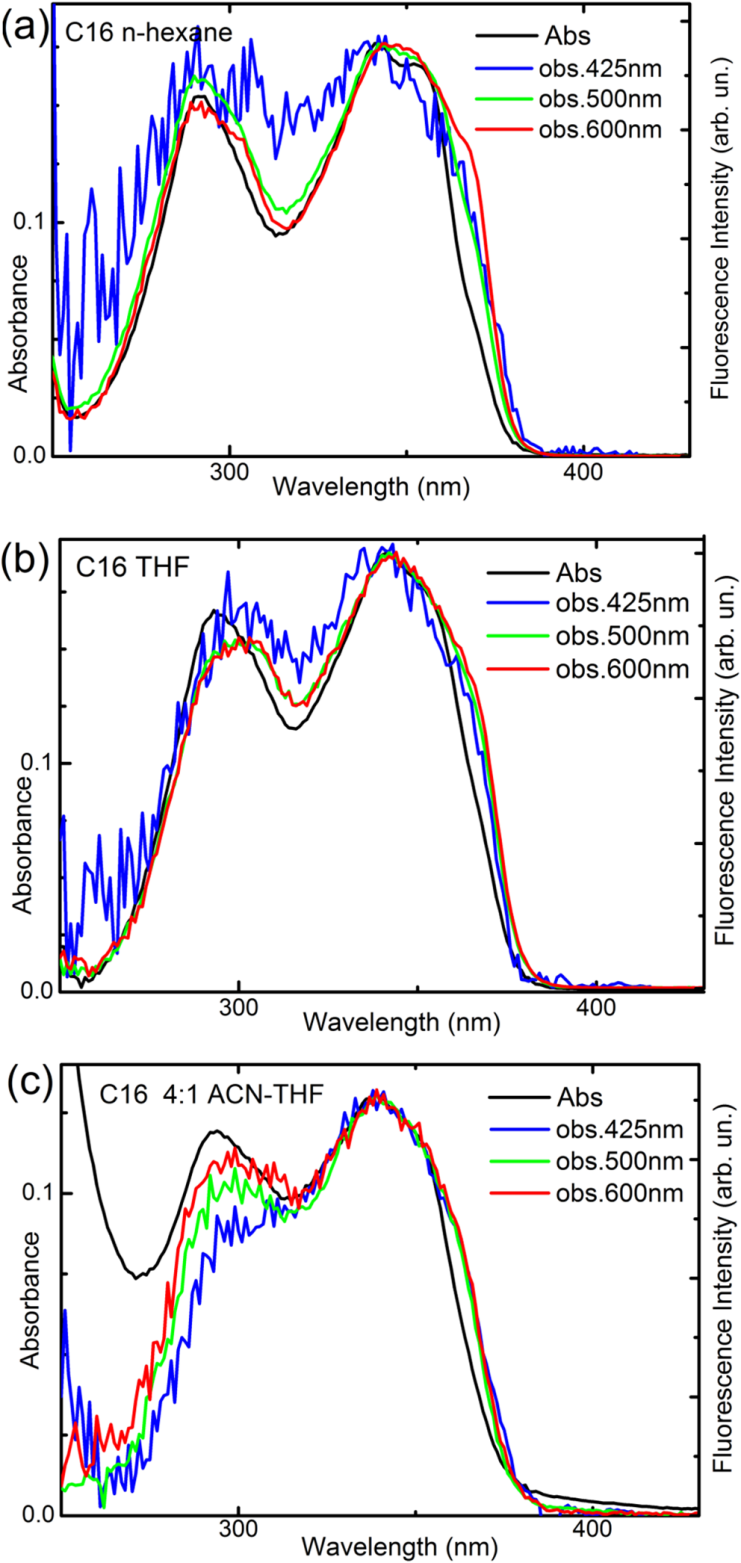
Comparison of normalized absorption (black line) and fluorescence excitation spectra of **C16** recorded at different wavelengths (425 nm—blue, 500 nm—green, and 600 nm—red lines) in (a) *n*-hexane, (b) THF, and (c) 4:1 ACN—THF mixture.

The emission spectrum obtained in *n*-hexane and *n*-hexadecane consists of a broad band with a maximum at 537 nm (Figure 3). In all solvents, the fluorescence spectrum of **C16** does not depend on the excitation wavelength (Figures S4 and S5). Also, the fluorescence excitation spectrum is independent of the observation wavelength, and its shape is similar to that of the absorption spectrum (Figure 4), demonstrating that the emission arises solely from a single species, with no contribution from impurities or other conformations. The observed Stokes shift for **C16** is of the same magnitude as previously reported for TSALs (9679 cm^–1^).^36^ This suggests that the relaxation process involves a significant conformational rearrangement in the excited state. Based on the kinetic results presented in the next section, this process may be attributed to ESIPT occurrence. Notably, the fluorescence of **C16** in *n*-hexane is nearly pure ESIPT emission. In the more viscous *n*-hexadecane, the same pattern is observed, with the residual locally excited (LE) state band exhibiting a very low intensity (less than 1%) compared to the ESIPT emission band, and this intensity is much lower than in TSANs.^17,18^

Thus, this is the first reported example of TSAL showing nearly pure ESIPT emission in *n*-alkanes. In toluene and tetrahydrofuran (THF), a weak LE band is distinguishable, though its intensity remains small relative to that of the ESIPT band (around 5%). The LE band intensity increases with the solvent’s dielectric constant. In polar mixtures of THF and acetonitrile (ACN), a blue-shifted shoulder is discernible in the emission spectra. Additionally, the decay time of the LE state increases, as does the growth time of the ESIPT band (Table S6). Such findings may be linked to the inverted Marcus region, where increased solvent polarity decreases the rate of excited-state proton transfer (ESIPT), reducing its prevalence and allowing the population of the LE state increase upon excitation. Additionally, the formation of intermolecular hydrogen bonds between the chromophore and protic solvent molecules surrounding the proton transfer sites, as suggested by microsolvated **Me** with three methanol molecules (Figure S2), would further decrease the ESIPT/LE ratio. The fluorescence profile observed in methanol is dominated by LE emission; however, its broad and intense red tail suggests the possibility of an ESIPT occurrence (Figure 3). Indeed, the intensity of the latter increases significantly in less protic 1-octanol and more polar DMF solution, despite their polarity and proticity. The obtained spectra in protic solvents suggest that the formation of a hydrogen bond with the solvent introduces new degree of freedom for structural changes that may hinder the ESIPT process.

**Figure 5:**
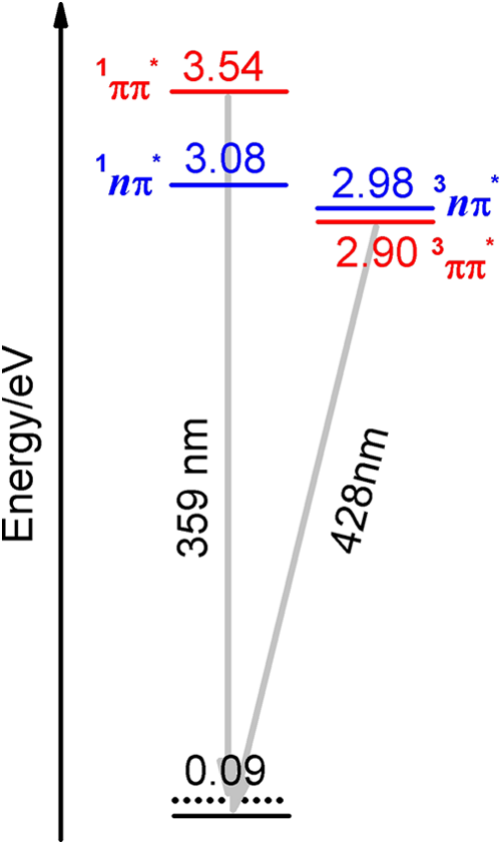
Energy diagram for **Me** computed at the MP2/ADC(2)/cc-pVDZ level of theory imposing the *C*_*s*_ symmetry to allow the separation of ππ* (red) and nπ* (blue) states. Gray arrows indicate the fluorescence and phosphorescence from ^1^ππ* and ^3^ππ*. Numbers denote relevant energy in eV.

Low-temperature emission spectra recorded at 77 K measured in *n*-hexane, methylcyclohexane, tetrahydrofuran, and DMF (Figure S6) reveal that no ESIPT is observed, proving the existence of a barrier for the proton transfer reaction. Additionally, phosphorescence is observed at low temperatures. This finding is in line with the near degeneracy of ^1^nπ* and ^3^ππ* states computed at the ADC(2)/cc-pVDZ level of theory, constraining the system to the *C*_*s*_ symmetry. Such proximity favor the intersystem crossing between them and consequent phosphorescent emission, as suggested by the El-Sayed rule.^37−39^ A simplified energy diagram is presented in Figure 5 below.

#### Kinetic Measurements

**Figure 6:**
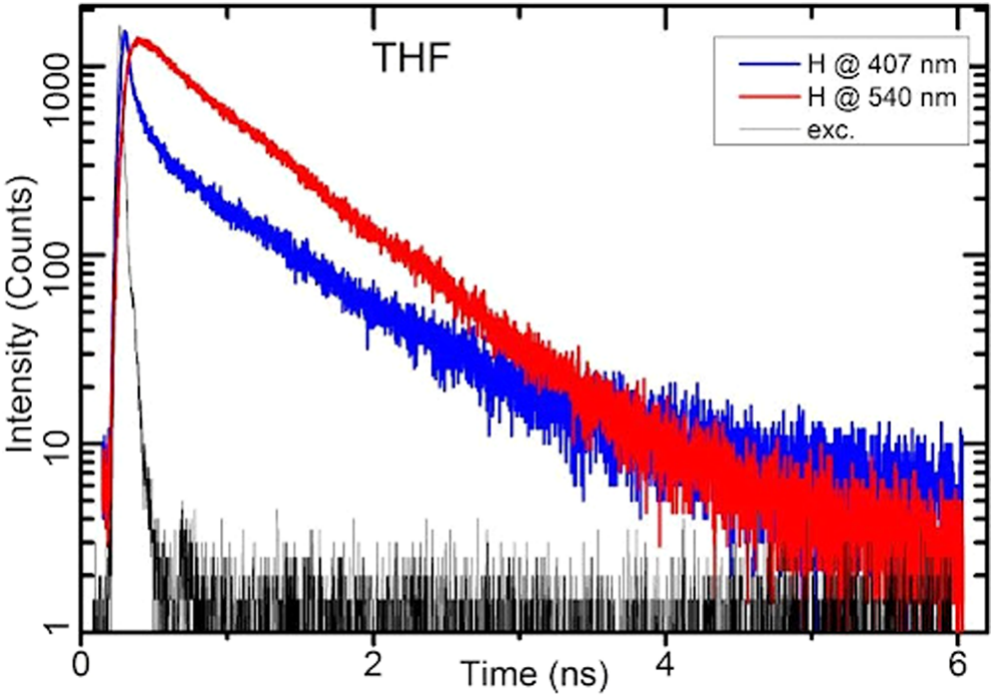
Fluorescence decay profiles of **C16** obtained in THF for LE emission recorded at 407 nm (blue) and ESIPT emission recorded at 540 nm (red line). “H” stands for the protonated form of **C16**. Excitation at 374 nm (black line).

The fluorescence quantum yield (FQY) of **C16** in *n*-alkanes is remarkably higher than that of previously studied TSALs.^17,18^ FQY decreases with the increment of the dielectric constant of the medium (ε) (Table 1), revealing the influence of the ESIPT state energy on the rate constant of photophysical processes. Moreover, the FQY is slightly higher in viscous *n*-hexadecane than in *n*-hexane, indicating that the reduction of large-amplitude motions decreases the rate of nonradiative processes, most likely internal conversion, which becomes effective when rotation and excited-state geometry changes are allowed. The changes in FQY correlate well with lifetime data (Table 1), proving that the rate of radiationless processes depends on the viscosity and *ε* values of the solvent environment. In order to elucidate mechanisms involved in the photophysics of **C16**, a kinetic investigation was performed by using a series of solvents. Fluorescence decay profiles were recorded at multiple wavelengths for each solution. Figures 6 and S7 depict selected kinetic profiles for LE and ESIPT emission. Figures S8–S13 present fits of multiexponential decays to fluorescence decay traces, whereas Tables S5–S9 show selected results obtained from the deconvolution program. In the fluorescence decay trace of **C16** in THF, both fast and long decay components are clearly visible (Figure 6). Deconvolution reveals three components with decay times of 18, 336, and 890 ps (Table S7). The kinetic profile of the ESIPT reaction exhibits distinct characteristics. Fluorescence intensity increases gradually at short times, reaching its maximum later than the LE emission and subsequently decays more rapidly than the LE trace. Deconvolution yields a growth time (component with negative amplitude) of 54 ps and two decay components with times of 75 and 679 ps. The sum of all amplitudes is close to zero, indicating that at time zero, the tautomer is not populated and a precursor-successor schema applies for both LE and ESIPT states. However, the mechanism is not simple as the fast decay time of LE differs from the growth time of ESIPT, suggesting that an intermediate or transition state may be involved in the proton transfer process. The existence of the third time supports this hypothesis. The different decay times of LE and ESIPT prove that both states are not in thermal equilibrium. The case of **C16** clearly differs from twisted TSALs with steric hindrance, for which the thermalization of LE and ESIPT states by fast forward and backward proton transfer was reported.^18^ The complexity of the **C16** case may be derived from the possibility of two processes: ESIPT and out of plane distortion involving the enaminic nitrogen and/or relevant conformational rearrangement upon excitation. Being not thermalized, both states are only once populated and then depopulated, allowing a rough evaluation of their relaxation rate constants. Splitting the total FQY, Φ, into fractional FQY of each state, Φ_LE_ and Φ_ESIPT_, and using decay times, one may calculate the radiative and nonradiative rate constants: *k*_r_ = Φ/τ and *k*_nr_ = (τ)^−1^ – *k*_r_. For the LE state, *k*_nr_ (and thus the rate constant of the ESIPT process that dominates the relaxation) decreases with the solvent dielectric constant (Table S6). A similar finding was reported for other TSALs. For the ESIPT state in solvents (except alcohols), the radiative rate remains constant, whereas *k*_nr_ increases with *ε* of the medium (Table S5). For solvents forming hydrogen bonds, the intensity of LE emission is comparable to that of ESIPT, and a strong overlap of both bands occurs (Figure 3). This, along with the appearance of new structured emission bands, may be the reason that no growth is observed in the kinetic profiles recorded for the red emission band in alcohols (Table S9). In DMF, the growth time was observed only at the very red part of the ESIPT band (Table S9). The formation of hydrogen bonds with solvent molecule(s) makes its photophysics more complex. Deuteration of **C16** affects the fast decay component time of LE and the growth time of ESIPT, making them both longer (Table S7). The apparent kinetic isotope effect (KIE = τ_D_/τ_H_) is always greater than one for the fast decay time of LE and growth time of the ESIPT kinetic profiles (Table S8). Long decay times of LE and ESIPT are not affected much by deuteration (Table S7), and their KIE parameter is about one (Table S8), pointing out that purely electronic factors are important for radiative and nonradiative relaxation processes in LE and ESIPT states. The characteristic time of the fast LE decay component containing rate constants of all relaxation processes, 1/τ_LE_ = *k*_r_ + *k*_isc_ + *k*_ic_ + *k*_ESIPT_, is rather long and always within the range of 20 to 80 ps, indicating a relatively slow ESIPT process. Growth times of ESIPT kinetic profiles on the order of 50 to 90 ps support this conclusion and suggest the existence of a barrier for proton transfer.

Our results published elsewhere^17,18,32^ suggest that the photophysical behavior of TSAL derivatives involves the population of multiple species in the excited state (tautomers and rotamers) due to the occurrence of the ESIPT reaction, besides on the Franck–Condon locally excited state. This characteristic leads to a broad fluorescence spectrum, in general with a low ESIPT/LE emission band intensity ratio. However, competing nonradiative channels through large-amplitude movements can significantly decrease the emission efficiency. Experimental measurements show that the barrier for the proton transfer reaction can be modulated by the solvent polarity and proticity since the tautomer is significantly more polar than the LE state. Not only the intensities of ESIPT and LE bands will be affected by electronic effects, but their positions and intensities may also be modulated through the insertion of bulky alkyl groups in the *ortho* position of TSANs, what constrain the rotation of aniline groups, suggesting that the rate of proton transfer depends on the twist angle and solvent viscosity. Remarkably, the inversion of the ESIPT/LE ratio for **C16** provides a more comprehensive understanding of the mechanisms involved in the luminescence of the TSALs.

It is worth noting that our results for **Me** and **C**_**16**_ can be classified as *kinetic* ESIPT, whereas the earlier studied **dH**, **dMe**, **dEt**, and **dPr** molecules^18^ are best described as exhibiting *thermodynamic* ESIPT.^40,41^

## Conclusions

A detailed analysis of the photophysical properties of **C16** was performed. Experimental measurements indicate that the ESIPT reaction occurs in **C16** in nonprotic solvents, consistent with previous findings for this class of compounds. However, the ESIPT/LE emission intensity ratio is inverted compared to that of the TSANs previously published in nonpolar solvents. This unprecedented behavior results in the nearly pure tautomer (*kke*) emission.

*Ab initio* (ADC(2)/cc-pVDZ) calculations performed on the model compound **Me** indicate that the experimentally observed ESIPT/LE inverted intensity emission ratio in nonpolar solvents may be related to nπ* – ππ* mixing. This behavior would be enhanced by the Jahn–Teller effect due to the degeneracy of bright states in **Me**, allowing for the population of a species with an *N*-pyramidalized proton transfer site, resulting in an out of plane structure. This process is expected to compete with LE emission, which quenches its fluorescence. Conversely, in protic solvents, the planar conformation is favored and a direct correlation between its emission and the increase of the LE band is observed. In protic solvents, the stabilization of distorted **Me** structures due to the formation of solute–solvent H-bonds is predicted to compete with the population of tautomers through the ESIPT process, facilitating access to the conical intersection with the ground state and effectively quenching the tautomer emission. Presented results reveal the complexity of TSALs photophysics. In particular, the unprecedented emission spectrum pattern of **Me** may arise from an indirect pathway that promotes the population of tautomer species by quenching the LE emission through the enaminic *N*-pyramidalization.

## Supporting Information Available

Detailed experimental procedures, characterization data, and the theoretical calculations (PDF)
