# Supplementary material for: Exploring Emission Ratios: Influence of Neighboring Groups on TSAL Core
Source: J Phys Chem A. 2025 Mar 27;129(14):3264–71. doi: 10.1021/acs.jpca.5c01560 (PMC12013964; doi:10.1021/acs.jpca.5c01560)
Supplement: Supplementary file 1 — jp5c01560_si_001.pdf [file jp5c01560_si_001.pdf]

Electronic Supplementary Information (ESI) for:

Exploring Emission Ratios: Influence of Neighboring Groups on TSAL Core

Olaf Morawski*, Pawel Gawrys, Marzena Banasiewicz and Cristina A. Barboza*
Institute of Physics, Polish Academy of Sciences, Al. Lotników 32/46, 02-668, Warsaw, Poland

* *E-mail: morawo@ifpan.edu.pl, crissetubal@ifpan.edu.pl*

1. Materials and methods

1.1. Synthesis

All chemicals and solvents were purchased from Aldrich or Fluorochem with the exception of the 1,3,5-triformylphloroglucinol which was purchased from Carbosynth. All reactants were used as received. None of the reactions and purifications required oxygen, water and ambient light exclusion.

1.2 Procedure and analytical data

Synthesis of **C16**. (2*E*,4*E*,6*E*)-2,4,6-*tris*[(n-hexadecylamino)methylidene]cyclohexane-1,3,5-trione (C_{3h} diastereoisomer) and (2*Z*)-2,4,6-*tris*[(*n*-hexadecylamino)methylidene]cyclohexane-1,3,5-trione (C_s diastereoisomer). In a one-necked flask (100 ml) was put 2,4,6-triphormylophloroglucinol (0.21 g, 1 mmol) and *n*-hexadecylamine (0.97 g, 4 mmol) and 2-propanol (p.a. grade, 40 ml). The mixture was gently refluxed with stirring for an hour. During that time the mixture became bright yellow solution. After that time the presence of two products was detected by thin layer chromatography (The reaction mixture was diluted with acetone and the TLC run in dichloromethane. The spots were visualized with a UV lamp). The mixture was next evaporated to dryness and methanol was added (p.a. grade, 75 ml). After one hour of vigorous stirring at ambient temperature the resulting suspension was filtered off on a sinter funnel (G3, 1 inch wide) and washed with methanol (p.a. grade, 3 x 30 ml, ambient temperature). The pale beige amorphous solid (0.80 g, 91%) was crystallized by quick dissolving in 2-propanol (Uvasol, 30 ml) and diluting with methanol (Uvasol, 60 ml, ambient temperature). The flask was placed in the freezer (-25°C) overnight. The next day the solids were filtered off a sinter funnel (G3, 1 inch wide) and washed with methanol (Uvasol, 3 x 30 ml, ambient temperature). After 24 hours of drying in air, the product was dried in a vacuum desiccator for 48 hours (ambient temperature, 2 mbar). Off-white semi-crystalline solid (738 mg, 84%). (preferably overnight). The product has high affinity to the polar and protic solvents. ^1H NMR analysis indicates that the compound exists in the form of two isomers. The product does not stain TLC plates and the two spots can be only visualized with a UV lamp. HRMS (ESI^+): calculated ($M+H^+$): 880.8229, found: 880.8236, error: 0.79 ppm. HRMS (ESI^+): calculated ($M+Na^+$): 902.8048, found: 902.8049, error: 0.11 ppm. HRMS (ESI^-): calculated ($M-H^-$): 878.8083, found: 878.8090, error: 0.80 ppm. ^1H NMR ($CDCl_3$ + TMS, 298 K, 500 Mhz): 11.40 (multiplet, $J_{keto\text{-}enaminic}$ = 13.4 Hz, J_{alkyl} = 6.7 Hz, N-H proton of the C_s diastereoisomer, this signal obscures the signal from the N-H protons of the C_{3h} diastereoisomer), 10.98 (multiplet, $J_{keto\text{-}enaminic}$ = 13.4 Hz, J_{alkyl} = 6.3 Hz, signal

resulting from two partially overlapping multiplets of the N-H protons of the C_s diastereoisomer), 8.25 (doublet, $J_{keto\text{-}enaminic}$ = 13.5 Hz, =C-H proton of the C_s diastereoisomer), 8.23 (doublet, $J_{keto\text{-}enaminic}$ = 13.7 Hz, =C-H proton of the C_s diastereoisomer, partially overlaps with the signal at 8.25 ppm), 8.15 (doublet; $J_{keto\text{-}enaminic}$ = 13.5 Hz, =C-H proton of the C_{3h} diastereoisomer), 8.11 (doublet, $J_{keto\text{-}enaminic}$ = 13.6 Hz, =C-H proton of the C_s diastereoisomer, partially overlaps with the signal at 8.15 ppm), 3.34-3.42 (multiplet resulting the overlap of four triplets, J_{alkyl} = 6.6 Hz, 6H, alkyl N-CH_2-), 1.65 (apparent sextet, J_{alkyl} = 6.7 Hz, 6H, alkyl CH_2, overlaps with the residual water peak), 1.20-1.40 (multiplet, 78H, alkyl chain CH_2), 0.88 (apparent triplet, J_{alkyl} = 6.7 Hz, 9H, alkyl CH_3). IR (KBr, cm^{-1}): 2956, 2918, 2872, 2850, 1624 (C=O), 1600, 1545, 1470, 1456, 1385, 1328, 1302, 1270, 1250, 1232, 1224, 1208, 1189, 1174, 1150, 1131, 1110, 1092, 1070, 1049, 1029, 1000, 835, 767, 736, 720. Elemental analysis: ($C_{57}H_{105}N_3O_3$), calculated: C(77.76%) H(12.02%) N(4.77%) O(5.45%), found: C(76.68%), H(11.27%), N(5.23%). Melting point: (2-PrOH:MeOH 1:2): 89-90 ºC.

1.3. Instruments and techniques

^{1}H FT-NMR spectra were recorded using Bruker AVANCE 500 MHz spectrometer. The chemical shifts are given vs. TMS as an internal standard. The coupling constants were calculated by using chemical shifts reported in the Hz unit (ChemSketch 12.0). FT infrared spectra were recorded using Bruker Vertex v80 FT-IR spectrometer. High resolution mass spectra (ESI-TOF-MCP) were obtained with a Shimadzu LCMS-9030 mass spectrometer. Melting points were obtained with an A. KRÜSS Optronic KSP1N melting point meter. Elemental analysis was conducted with an Elementar Vario EL III analyzer. Thin layer chromatography (TLC) analysis was carried out using Merck Silica gel 60 F 254 TLC plates and spots were visualized under UV light (365 or 254 nm).

For the optical measurements at ambient temperature (21 °C), spectroscopic grade solvents (Merck-Uvasol or Sigma-Aldrich HPLC grade) were used as received. Room temperature measurements were performed with dilute solutions in standard quartz cells (10 × 10 mm). Absorption spectra at 21°C were recorded using a PerkinElmer Lambda 35 spectrophotometer. Emission spectra at room temperature (21°C) were obtained using a FLS 1000 spectrofluorimeter, Edinburgh Instruments. The fluorescence kinetics studies were performed using the time correlated single photon counting technique with 2.04ps per channel temporal resolution. A mode-locked Coherent Mira-HP picosecond laser pumped by a Verdi 18 laser was used for excitation. The fundamental of the Mira laser (750 nm) was up converted to 375 nm. The temporal width of the excitation pulses was about 180 fs and of the instrument response function (IRF) about 50 ps Fluorescence was dispersed with a 0.25 m Jarrell-Ash monochromator and detected with an HMP-100–07 hybrid detector coupled to an SPC-150 PC module, (Becker&Hickl GmbH). Fluorescence decays were analyzed with a deconvolution software using a nonlinear least squares procedure with the Marquardt algorithm. A standard χ^2 test along with residual and autocorrelation function plots were used to assess the quality of a fit. The estimated accuracy for the determination of decay time was about 10 ps.

In order to evaluate the effect of rigidity of the matrix, the photophysical properties were studied in frozen *n*-hexane, MCH, THF and DMF solutions at a temperature of 77 K and using the viscous *n*-hexadecane at ambient temperature (21°C). The liquid solutions were poured into fused silica NMR tubes, degassed and quickly cooled in liquid nitrogen (77 K) to prevent

aggregation. The low-temperature spectra of solutions in tubes inserted into liquid nitrogen dewar with optical windows of Edinburgh Instruments were recorded also with FLS 1000 spectrofluorimeter.

2. Synthesis and characterization

The synthesis of tris(salicylidenealkylamine) bearing *n*-hexadecyl substituent (**C16**) is presented in Scheme 1. **C16** was trivially synthesized by the condensation reaction between 2,4,6-triformylphloroglucinol[1] and a slight excess of *n*-hexadecylamine as an only step. Due to the long alkyl chains in its structure, **C16** is easily precipitable and crystallizable from alcohols. The compound has quite good solubility in chloroform (~20 mg/ml) and sufficient solubility in other solvents for our photophysical studies with the exception of acetonitrile (complete insolubility).

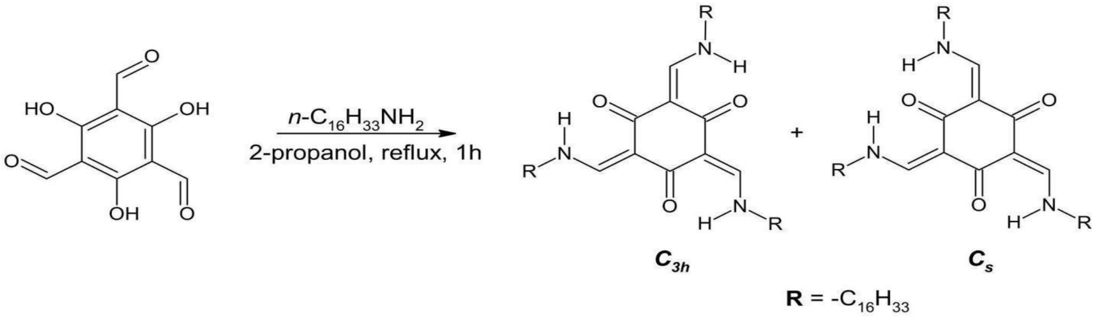

Scheme S1. The synthesis of two diastereoisomers of **C16**, where the *cis-kkk* and *trans-kkk* species correspond to the C_{3h} and C_s point group, respectively.

^{1}H NMR spectroscopy shows that **C16** exists almost solely as an asymmetric C_s isomer. From the integration values of the diagnostic NMR resonances, it was estimated that for **C16** the C_{3h}:C_s ratio is approximately 1:20, thus the symmetric isomer consists of at most 5% of the diastereoisomeric mixture. The position and multiplicity of proton resonances prove that **C16** also adopts *keto-enamine* form in the ground state, like practically all TSANs with aryl substituents. The chemical shifts on keto-enamine NH protons of **C16** (<12 ppm) are much smaller than for *N*-aryl counterparts. Also the peaks are no longer appearing as doublets as there is an additional coupling from the N-CH_2-R alkyl protons, as easily proved by ^{1}H-^{1}H COSY NMR spectroscopy. Still the large coupling constant of 13.4 Hz, between NH and CH enamine protons is observed. The latter, that is, vinylic protons of the *keto-enamine* group remain doublets within the range of 8.10-8.25 ppm and the coupling constant of 13.4 Hz. Triple *N*-alkyl keto-enamine systems are rarely described. A structurally related *N*-decyl TSAL has been disclosed previously[2] and expectedly it possesses very similar NMR characteristics. TSALs with chiral cyclohexyl substituents have also been reported.[3] **C16** is a very stable compound, and it can be stored in an airtight container with light exclusion for many years. Also, no signs of

1 S. H. M. Mehr, H. Depmeier, K. Fukuyama, M. Maghamia, M. J. MacLachlan, Formylation of phenols using formamidine acetate, *Org. Biomol. Chem.*, **2017**, 15, 581-583, https://doi.org/10.1039/C6OB02727J.

2 C. V. Yelamaggad, A. S. Achalkumar, D. S. S. Rao, S. K. Prasad , Self-Assembly of C3h and Cs Symmetric, Keto-enamine Forms of Tris(N-salicyl-ideneanilines) into Columnar Phases: A New Family of Discotic Liquid Crystals, *J. Am. Chem. Soc.*, 2004, **126**, 6506-6507, https://doi.org/10.1021/ja0495967.

3 P. Kieryk, J. Janczak, J. Panek, M. Miklitz, J. Lisowski, Chiral 2 + 3 Keto-Enamine Pseudocyclophanes Derived from 1,3,5-Triformylphloroglucinol, *Org. Lett.*, **2016**, 18, 12-15, https://doi.org/10.1021/acs.orglett.5b02989.

decomposition were noted when the analyzed samples lied in vials exposed to ambient light for several weeks. In contrast, tris(salicylidenealdimines) derived from dialkyl secondary amines no longer can form intramolecular hydrogen bonds and quickly undergo decomposition in air.[4] Infrared spectroscopy confirms that the molecules of **C16** possess a carbonyl group and long alkyl chains. The carbonyl stretching band is located at 1624 cm^{-1}. The band at 720 cm^{-1} is likely to be ascribed to the rocking terminal methyl groups band (ρ), as it is only noted for molecules possessing longer alkyl chains.

In the case of **Me**, the reaction was carried out accordingly, however, its isolation and purification was tedious. **Me** is an amorphous yellow solid, whereas **C16** is crystalline. Hence, **C16** was a significantly more reliable compound for optical studies. The presence of long fatty-type *n*-alkyl chains is expected to cause pronounced lipophilic interaction between the molecules. To assure that optical measurements of **C16** in non-polar solvents concern its monomeric species, concentration dependent fluorescence and excitation spectra of **C16** are included and some comparison spectra between **C16** and **Me** are listed below. A small 1 nm blue shift of **Me** in the absorption spectra results from the fact that methyl is a slightly weaker chromophore than longer alkyls.

---

4 S. H. M. Mehr , B. O. Patrick and M. J. MacLachlan , Stabilization of a Strained Heteroradialene by Peripheral Electron Delocalization, *Org. Lett.*, **2016**, 18 , 1840-1843, https://doi.org/10.1021/acs.orglett.6b00577.

3. Computational Studies

Table S1a. Vertical transition energy (ΔE), oscillator strength (*f*), dipole moment (*μ*), and leading electronic configurations of the *cis*-***kkk*** and *trans*-***kkk*** isomers of **Me** computed with the ADC(2)/cc-pVDZ method at the MP2/cc-pVDZ equilibrium geometry of the ground state.

State	ΔE\eV	f	μ/Debye	el. config.
		cis-***kkk***		
S_0	0.00	-	1.13	$(66a)^2$
$^3\pi\pi^*$	3.45	-	0.00	0.82(66a-67a)
$^3\pi\pi^*$	3.45	-	0.19	0.82(65a-67a)
$^3\pi\pi^*$	3.60	-	0.19	0.64(64a-67a)
$^1n\pi^*$	3.84	0.00	0.00	0.89(63a-67a)
$^1n\pi^*$	4.18	0.00	2.14	0.61(61a-67a)
$^1\pi\pi^*$	4.21	0.55	1.19	0.92(66a-67a)
$^1\pi\pi^*$	4.21	0.55	1.19	0.91(65a-67a)
$^1\pi\pi^*$	4.62	0.00	0.00	0.79(64a-67a)
$^1\pi\pi^*$	4.78	0.00	0.00	0.67(66a-68a)
		trans-***kkk***		
S_0	0.00	-	2.24	$(66a)^2$
$^1n\pi^*$	3.82	0.00	1.02	0.88(63a-67a)
$^1\pi\pi^*$	4.09	0.44	1.63	0.93(66a-67a)
$^1n\pi^*$	4.17	0.00	3.83	0.67(63a-68a)
$^1\pi\pi^*$	4.27	0.58	0.69	0.86(65a-67a)
$^1\pi\pi^*$	4.63	0.03	2.37	0.82(64a-67a)
$^1\pi\pi^*$	4.77	0.00	6.60	0.84(66a-68a)

Table S1b. Relevant molecular orbitals involved in the lowest electronic excitations of the *cis*-***kkk*** isomers of **Me** (cut-off = 0.02).

HOMO		*LUMO*	
65a	*66a*	*67a*	*68a*
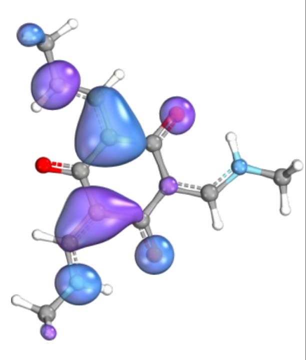	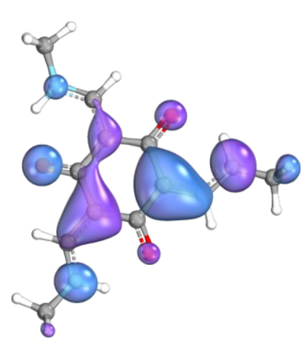	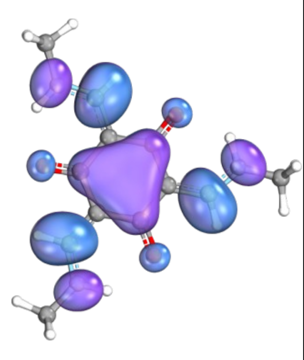	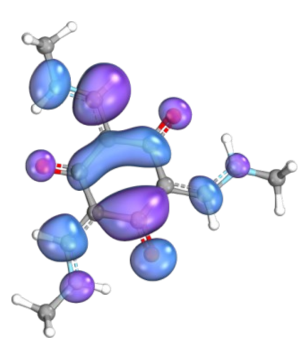

Table S2. Adiabatic energy of the fluorescing state (E(S_1)), vertical transition energy (ΔE), oscillator strength (f), and dipole moments ($\mu(S_1)$ and $\mu(S_0)$) computed for **Me** with ADC(2)/cc-pVDZ method at the equilibrium geometry of the S_1 state. Data computed with COSMO using dimethylformamide as solvent are given in parenthesis.

Isomer	*E(S₁)/eV*	*ΔE/eV*	*f*	*μ(S₁)/Debye*	*μ(S₀)/Debye*
cis-kkk (N-pyramidalized)	3.01	2.33 (2.38)	0.00 (0.00)	2.55 (3.66)	1.94 (2.32)
cis-kkk	3.60	3.38 (3.38)	0.40 (0.47)	1.41 (2.84)	0.27 (0.21)
trans-kkk	3.10	2.43 (2.43)	0.01 (0.01)	3.07 (3.85)	1.41 (2.03)
cis-kke	3.25	2.68 (2.55)	0.24 (0.22)	5.25 (6.34)	5.08 (4.68)

Table S3. The estimated overlap between bright ($\pi\pi^*$) and dark ($n\pi^*$) pairs of vertical states using the coefficients for configurations from each pair of states 1, 2, 3, and 4 of **Me**.

**Vertical States**		O_{ij}
1	2	0.63
1	3	0.78
1	4	0.80
2	3	0.34
2	4	0.65
3	4	0.78

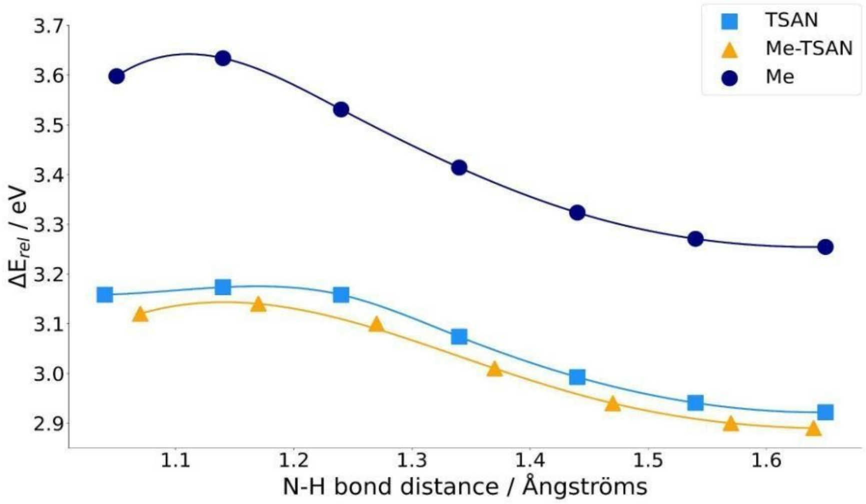

Figure S1. Minimum energy profiles for ESIPT reactions of **Me**, **Me-TSAN**, and **TSAN** obtained at ADC(2)/cc-pVDZ level of theory.

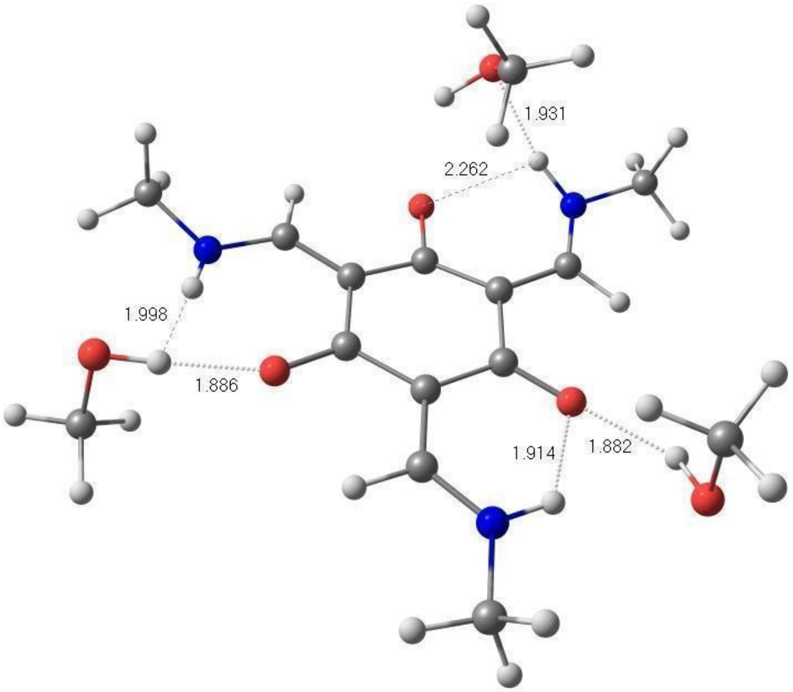

Figure S2. Intermolecular interactions between methanol molecules and **Me** proton-transfer sites, suggesting a competition among ESIPT occurrence and the formation of hydrogen bonds with protic solvent molecules neighboring proton transfer sites.

	kkk (N-pyramidalized)	***kkk (Planar)***	***kke***
**Me**	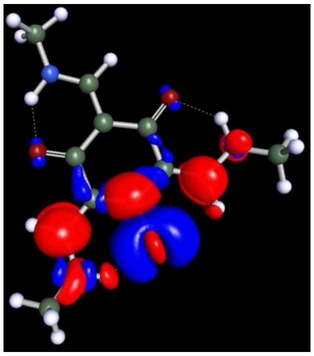	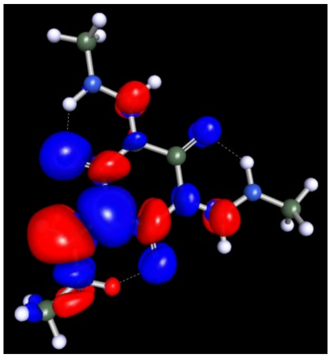	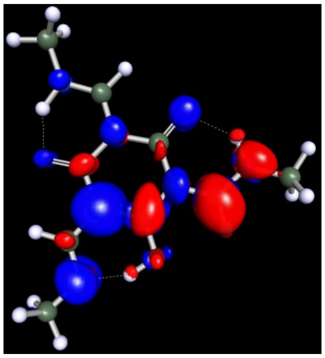
**TSAN-Me**		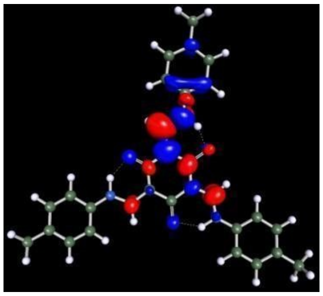	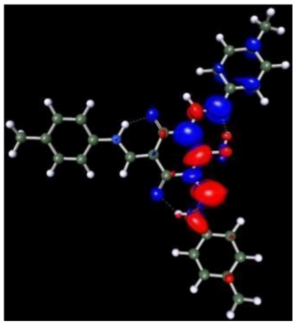
**TSAN**		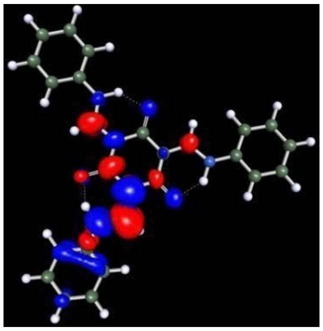	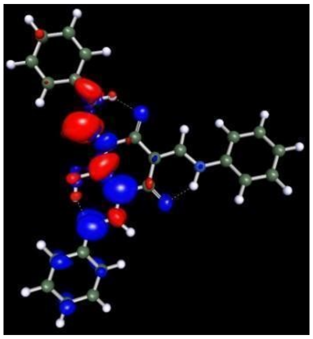

Figure S3. S_1-S_0 electron density difference computed for **Me**, **TSAN** and **TSAN-Me** at ADC(2)/cc-pVDZ level of theory (cut-off = 0.001) for ***kkk*** and ***kke***. Red (blue) indicates electron acceptor (donor) regions.

Table S4. Adiabatic energy of the fluorescing state (E(S_1)), vertical transition energy (ΔE), and dipole moments (μ(S_1) and μ(S_0)) were computed for low-lying singlet and triplet excited states. Geometry optimizations were performed using the ADC(2)/cc-pVDZ (ADC(2)/aug-cc-pVTZ) method for **Me** imposing C_s symmetry constraints.

State	***E(S₁)/eV***	***ΔE/eV***	***μ (S₁)/D***	***μ (S₀)/D***
$^1n\pi^*$	3.08 (3.20)	2.52	2.26	1.12
$^1\pi\pi^*$	3.55 (3.65)	3.45	1.47	0.33
$^3n\pi^*$	2.98 (3.14)	2.50	2.21	1.08
$^3\pi\pi^*$	2.90 (3.11)	2.92	0.18	1.54

4. Experimental results for C16

Measurements in powder phase

The fluorescence spectrum of **C16** in the powder phase is broad and structureless (Figure S14). Its shape depends on the wavelength of excitation, and fluorescence excitation spectra depend on the wavelength used for the measurement (Figure S15a and b). Both observations prove several states of different excitation energies are present in the powder. Grains of powder have different size, small volume and a relatively large surface, so it may be expected many electronic excited states of different energy may contribute to the emission spectrum. The luminescence spectrum is additionally influenced by reabsorption and energy transfer, which may alter its shape. Reabsorption reduces the blue-wing intensity, shortens its lifetime, and extends the lifetime of the red-wing. Energy transfer depopulates high-energy states, decreasing the blue-wing intensity, and populates low-energy states. This complexity does not allow to state that in powder the ESIPT process occurs as comparison of spectra in Figure S14 may suggest. At cryogenic temperatures, a strong phosphorescence spectrum is observed (Figure S15c and e), demonstrating the existence of an efficient intersystem crossing relaxation pathway. Fluorescence kinetic traces are multi-exponential, exhibit longer lifetimes in the red part of the spectrum, and indicate energy transfer between high- and low-energy states (Figure S16). Moreover, the very long emission observed on the red part of the spectrum (visible as the elevated background of the nanosecond decay profiles) suggests room-temperature phosphorescence. These findings would confirm the operation of efficient intersystem crossing (ISC), a conclusion derived already from the proximity of $n\pi^*$ and $\pi\pi^*$ states in 5K and 77 K spectra. The short lifetime, 297 ps (Table S5), also points to an efficient ISC process in the powder. Occurrence of proton transfer in the powder phase cannot be excluded; its presence, however, is masked by the complexity and variety of excited states and competition with energy transfer and ISC processes.

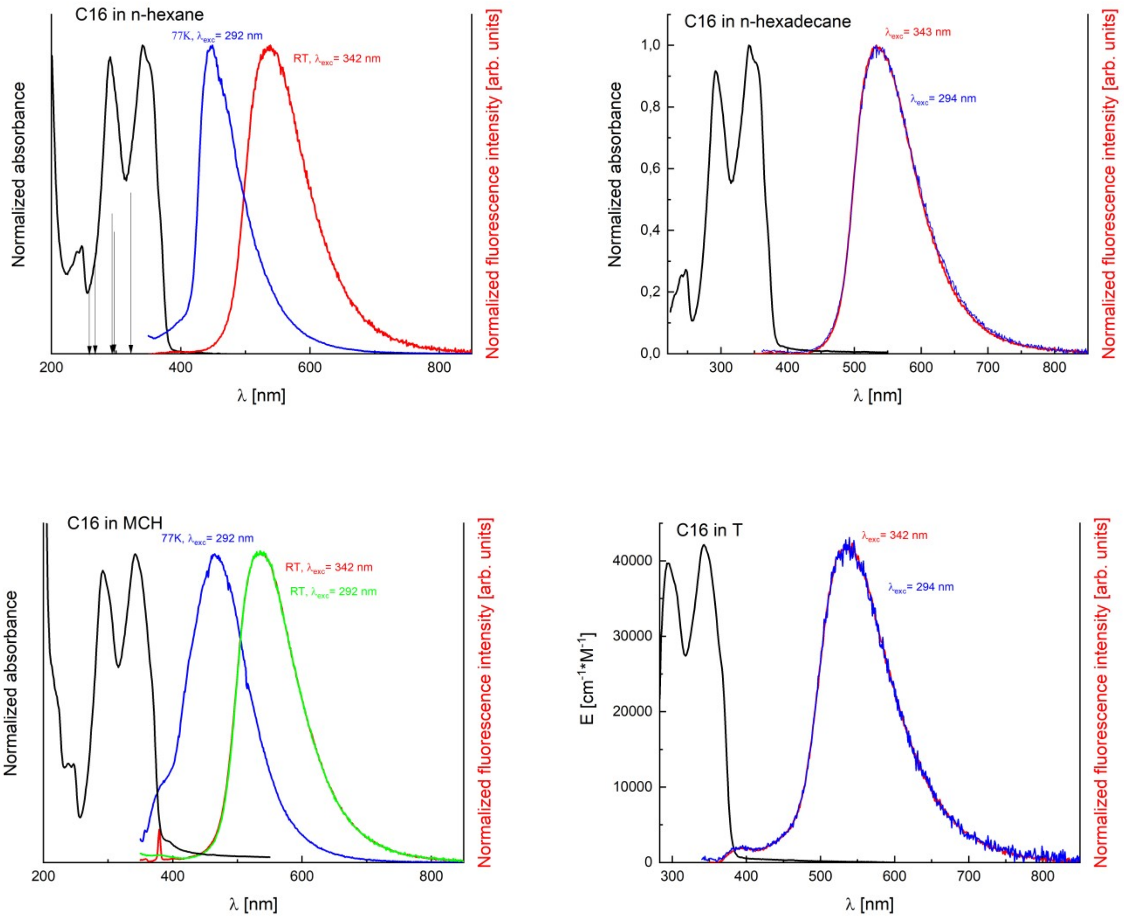

Figure S4. The absorption and emission spectra of **C16** in non-polar solvents (n-hexane, n-hexadecane, MCH - methylcyclohexane and T - toluene). The absorption spectra were recorded at RT, whereas for the emission spectra the excitation wavelengths and the temperature of the measurements are given.

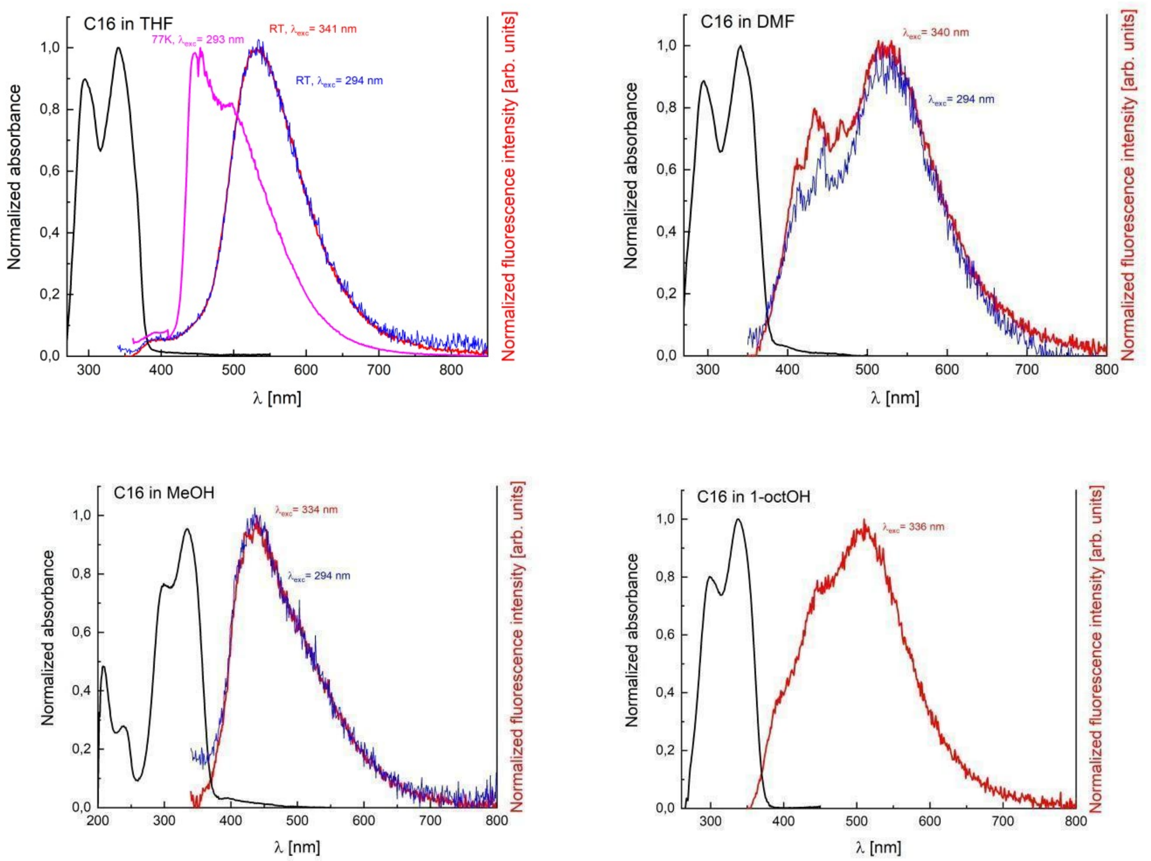

Figure S5. The absorption and emission spectra of **C16** in polar solvents (THF-tetrahydrofuran, DMF-dimethylformamide). The absorption spectra were recorded at RT, whereas for the emission spectra the excitation wavelengths and the temperature of the measurements are given.

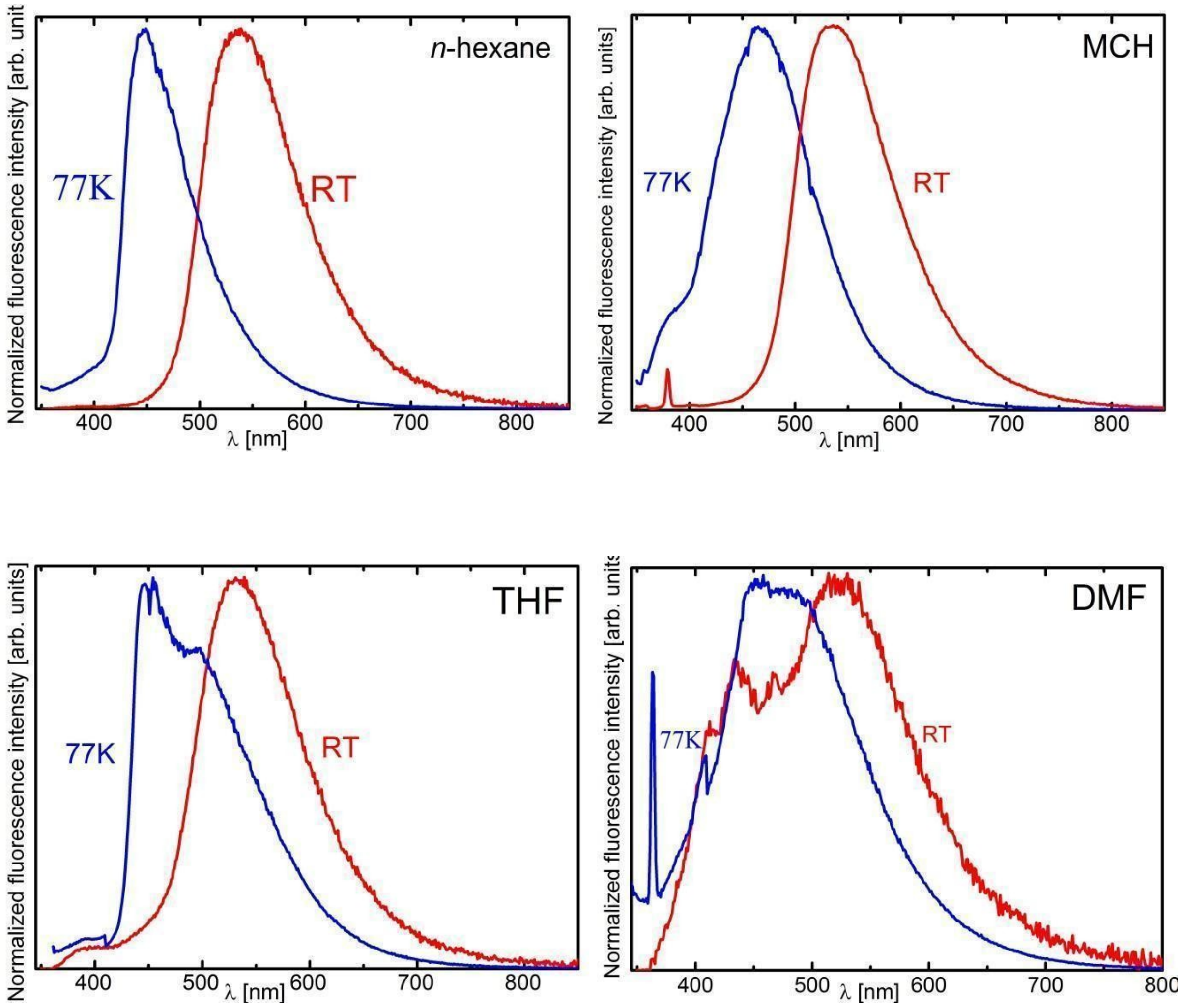

Figure S6. Emission spectra of **C16** in solvents at room temperature (red) and 77 K (blue lines).

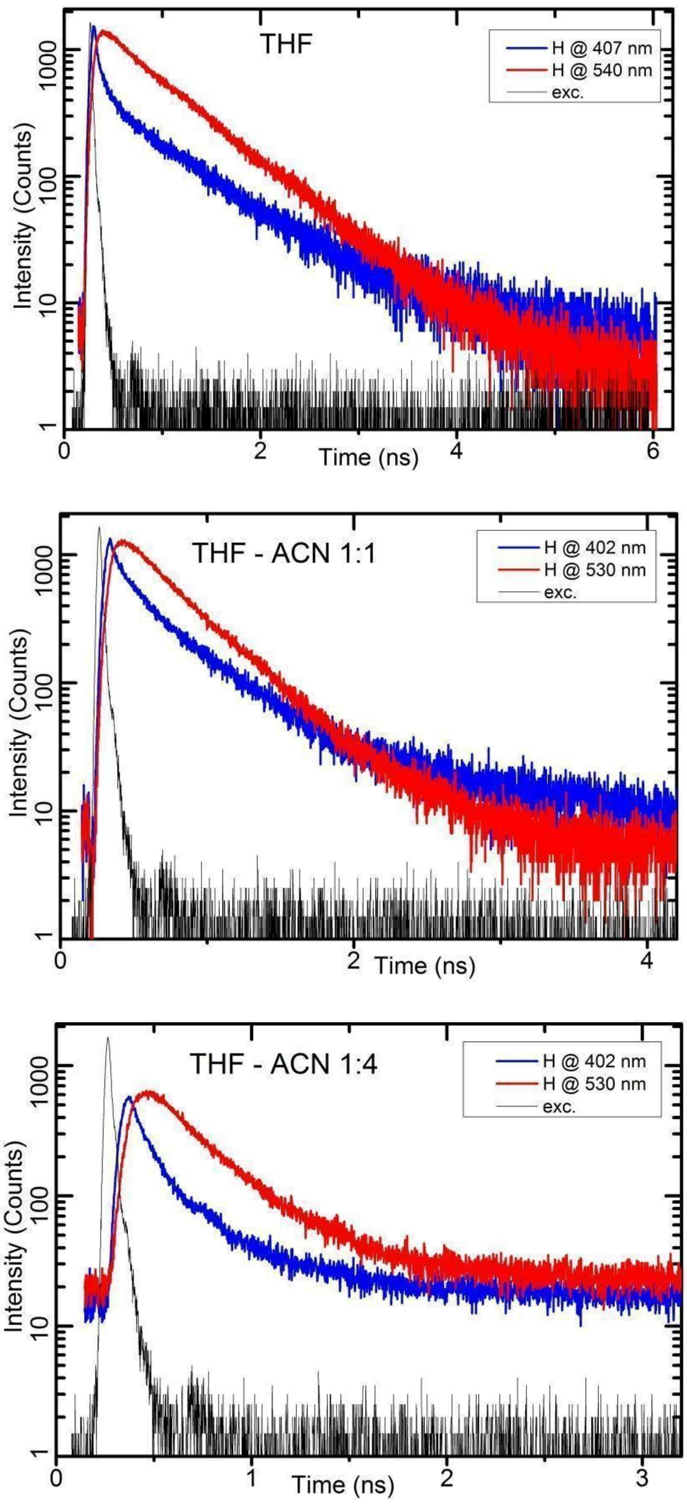

Figure S7. Fluorescence decay profiles of **C16** in THF and in THF with ACN mixture 1:1 and 1:4 v/v proportion. Legend specifies wavelength of observation. Letter "H" describes non-deuterated (proton) form of **C16**. Excitation at 374 nm.

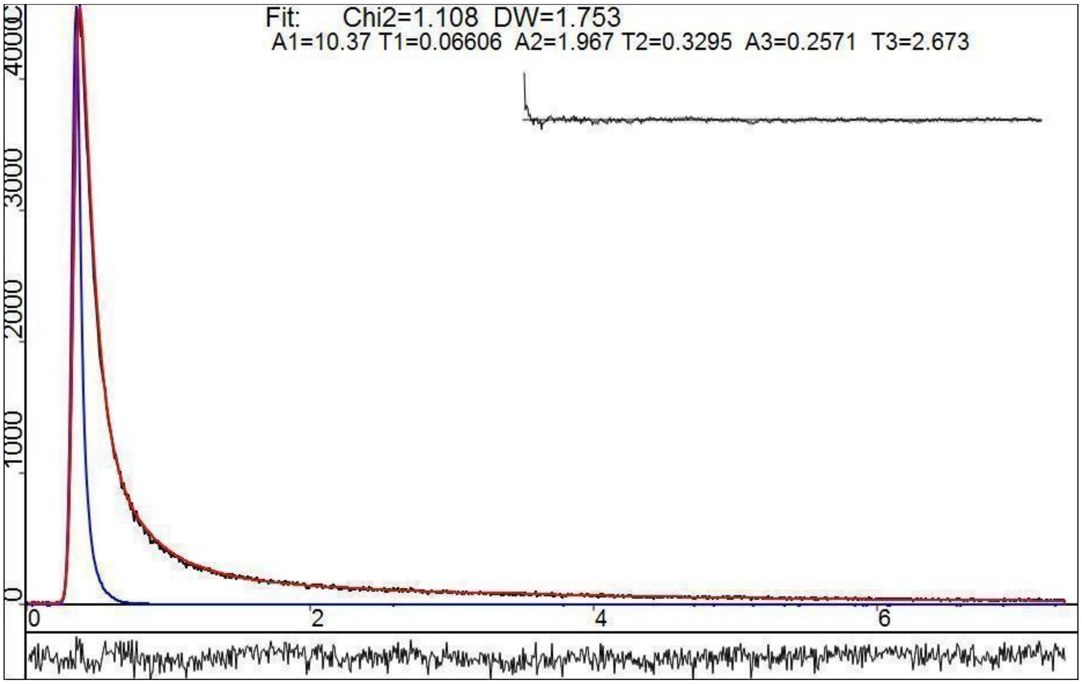

Figure S8. Fluorescence decay profiles of **C16** in 1-octanol recorded at 450 nm (black), decay profile obtained from deconvolution (red) and excitation pulse at 374 nm (blue). Below fit parameters the autocorrelation function is depicted (in black), whereas residuals are drawn below the experimental traces. Resolution 2.03 ps per channel.

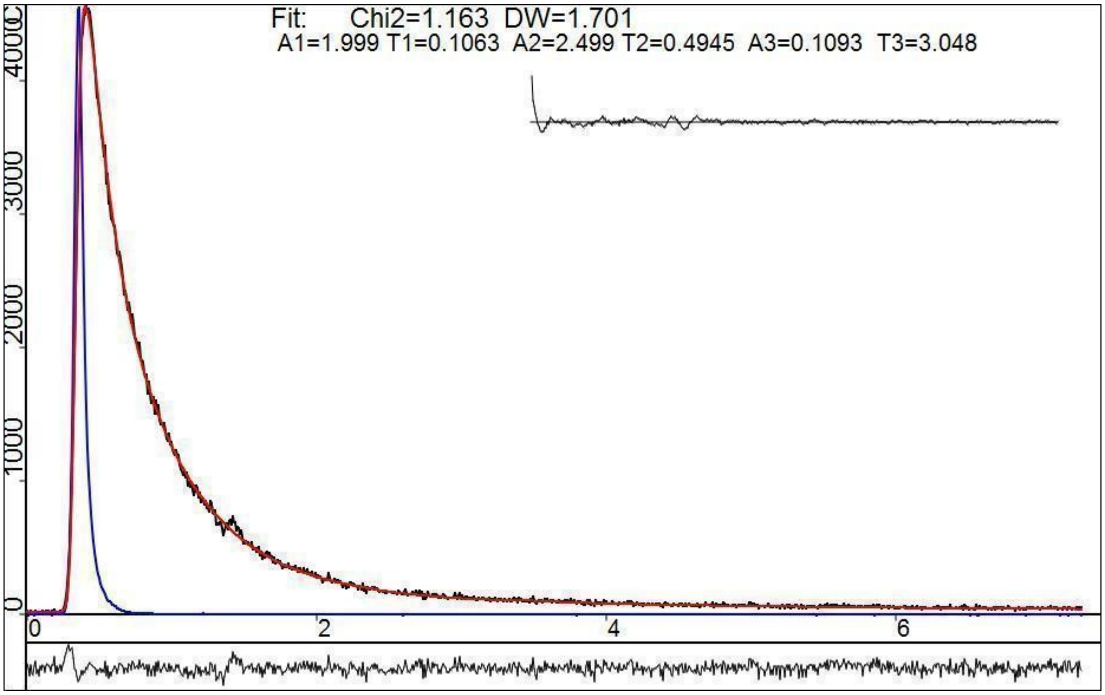

Figure S9. Fluorescence decay profiles of **C16** in 1-octanol recorded at 550 nm (black), decay profile obtained from deconvolution (red) and excitation pulse at 374 nm (blue).

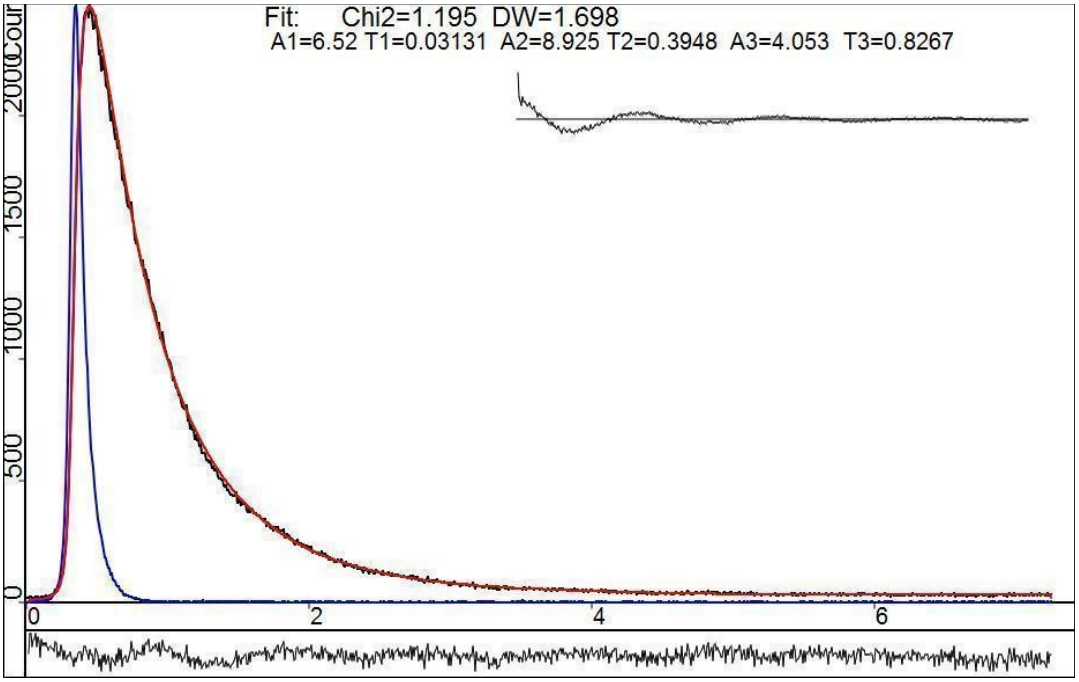

Figure S10. Fluorescence decay profiles of **C16** in methanol recorded at 437 nm (black), decay profile obtained from deconvolution (red) and excitation pulse at 374nm (blue). Below fit parameters the autocorrelation function is depicted (in black), whereas residuals are drawn below the experimental traces. Resolution 2.03 ps per channel.

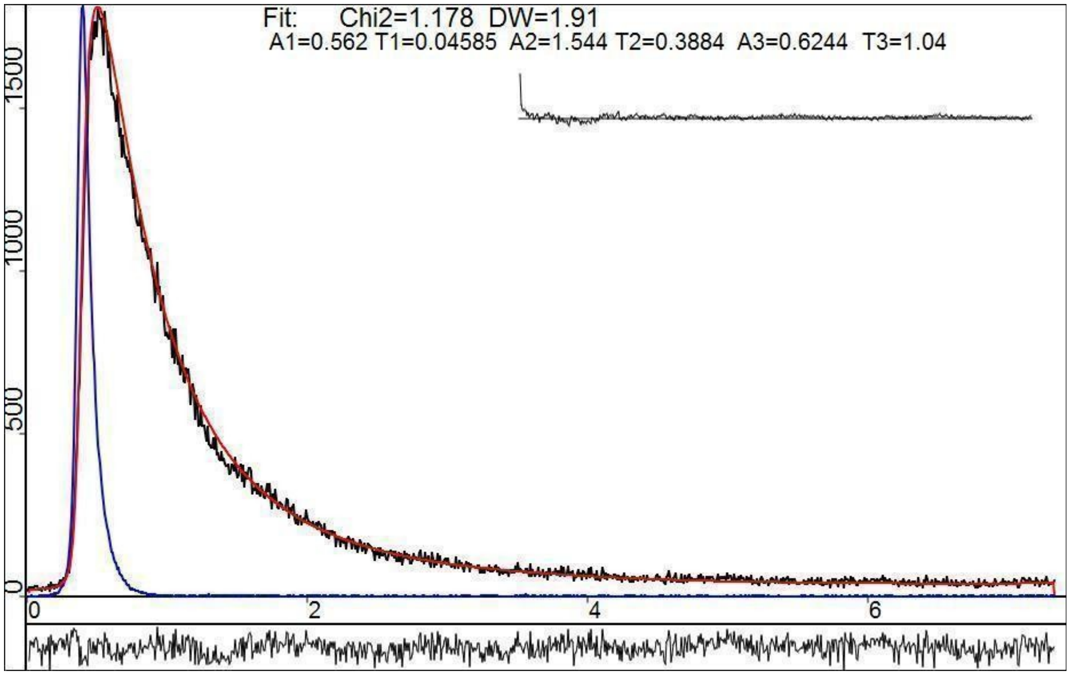

Figure S11. Fluorescence decay profiles of **C16** in methanol recorded at 550 nm (black), decay profile obtained from deconvolution (red) and excitation pulse at 374nm (blue).

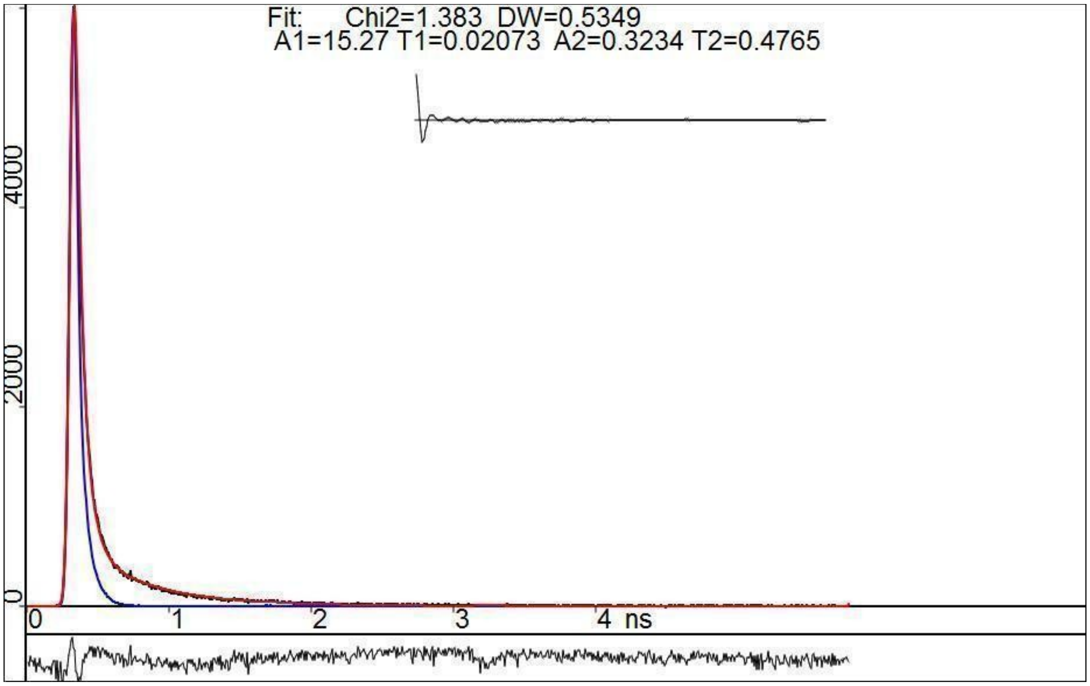

Figure S12. Fluorescence decay profiles of **C16** in DMF recorded at 390 nm (black), decay profile obtained from deconvolution (red) and excitation pulse at 374 nm (blue). Below fit parameters the autocorrelation function is depicted (in black), whereas residuals are drawn below the experimental traces. Resolution 2.03 ps per channel.

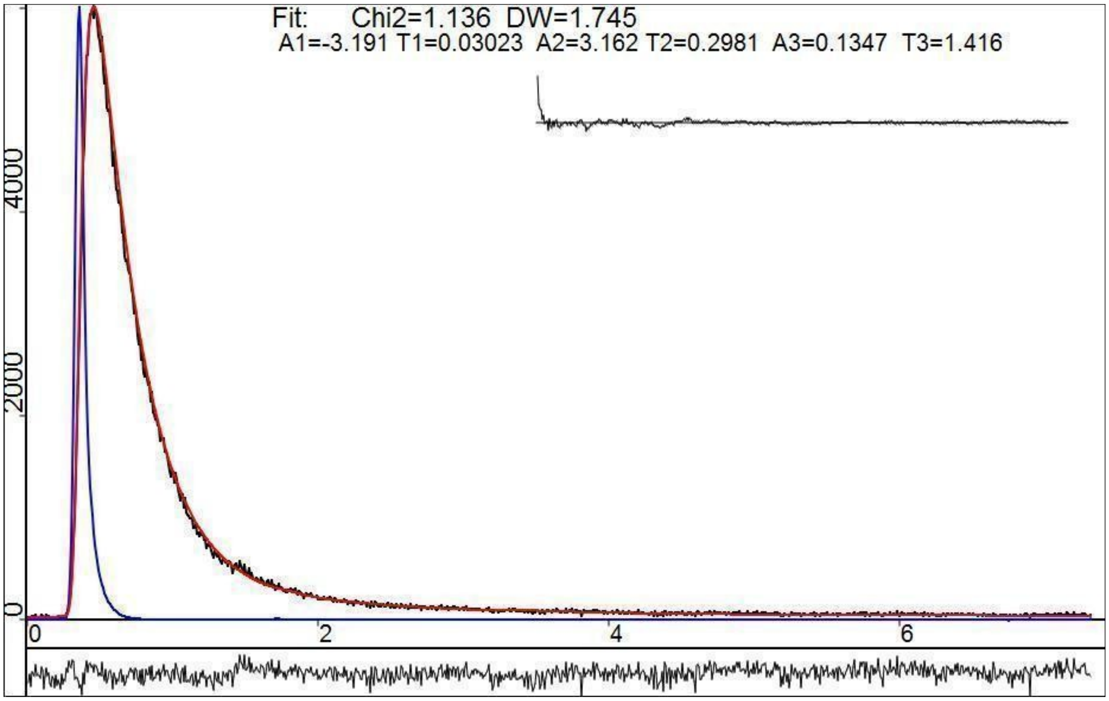

Figure S13. Fluorescence decay profiles of **C16** in DMF recorded at 560 nm (black), decay profile obtained from deconvolution (red) and excitation pulse at 374 nm (blue).

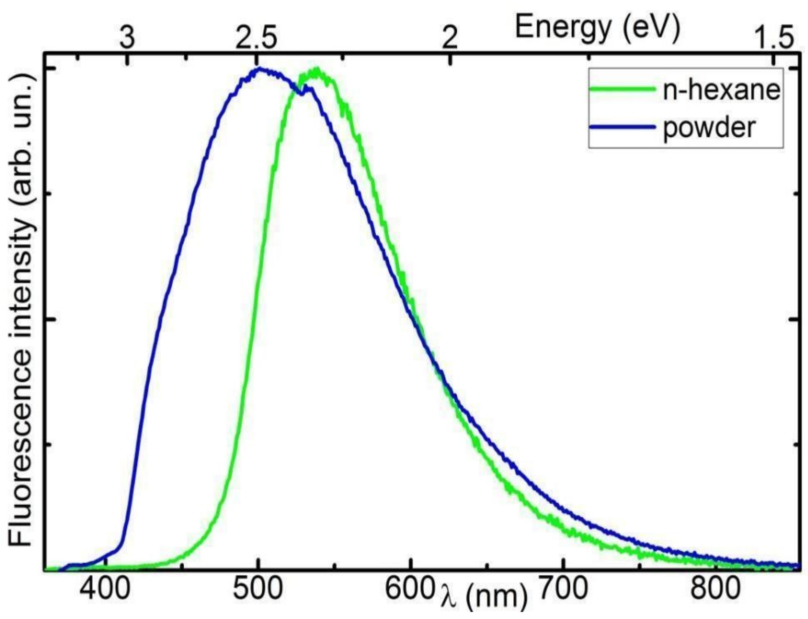

Figure S14. Fluorescence spectrum of **C16** in *n*-hexane (green line) and in powder phase (blue line) and recorded at room temperature with excitation at 294 and 340 nm respectively.

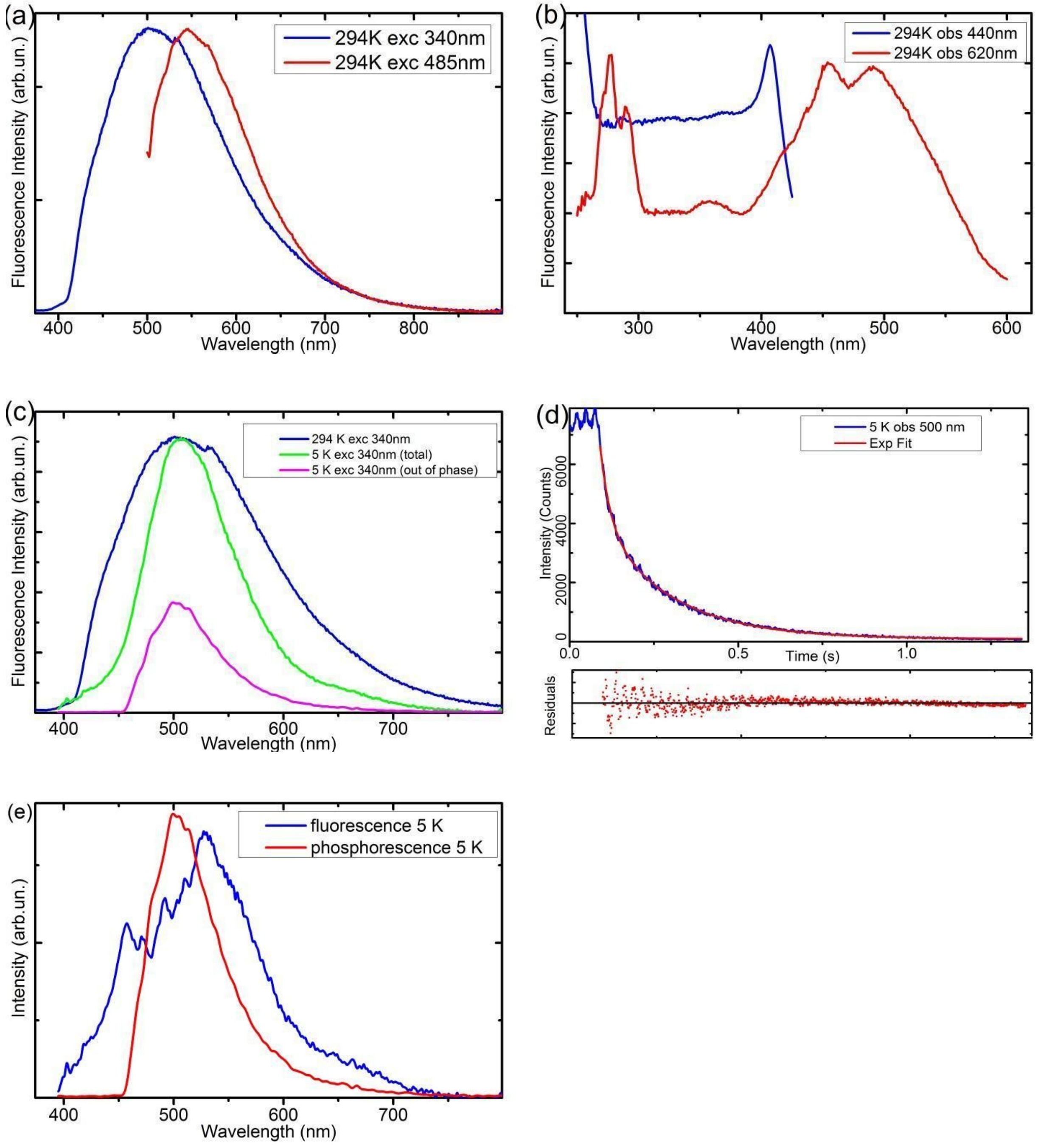

Figure S15. **C16** in powder phase: (a) fluorescence spectrum at 294 K excited at 340 and 485 nm, (b) fluorescence excitation spectrum at 294 K observed at 440 and 620 nm, (c) comparison of RT fluorescence with luminescence at 5 K – prompt (fluorescence + phosphorescence, green line) and delayed emission (phosphorescence, magenta line), (d) phosphorescence decay trace recorded at 500 nm and 5 K (blue) and exponential fit (red line) along with regular residual (bottom). The obtained phosphorescence decay time τ_{Ph} = 208 ±2 ms. (e) fluorescence and phosphorescence spectra obtained from “total” and “out of phase” spectra of (c).

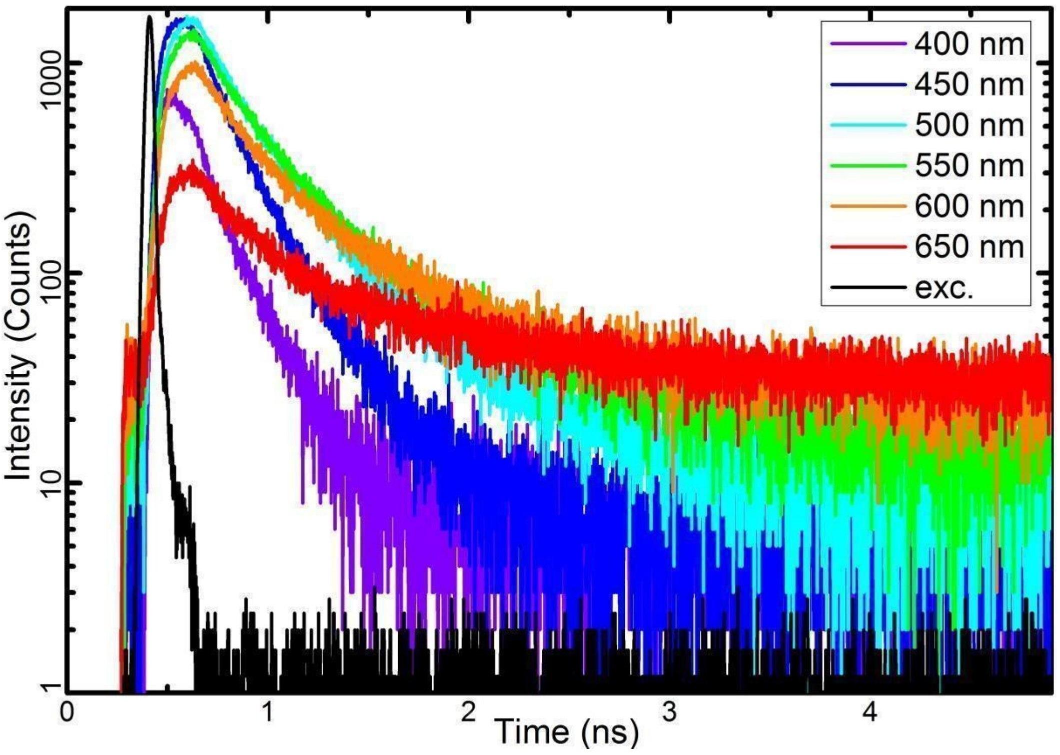

Figure S16. Fluorescence decay profiles of **C16** in powder obtained at room temperature with excitation at 374 nm. Legend defines the wavelength of observation.

Table S5. Decay time of the fluorescence red band (in most cases assigned to ESIPT), **τ**, and quantum yield, $\mathbf{\Phi}_{ESIPT}$, as well as total, $\mathbf{k_{tot}}$, radiative, $\mathbf{k_r}$, and nonradiative, $\mathbf{k_{nr}}$, rate constant for ESIPT band of the fluorescence spectrum of **C16** in solvents of dielectric constant **ε** and in powder.

Solvent	ε	τ / ns	Φ_{ESIPT} / %	k_{tot} /ns^{-1}	k_r /ns^{-1}	k_{nr} /ns^{-1}
n-hexadecane	2.05	1.358	2.9	0.736	0.021	0.715
n-hexane	1.89	1.334	2.6	0.750	0.020	0.730
Toluene	2.38	0.72	1.5	1.389	0.021	1.368
THF	7.6	0.677	1.08	1.477	0.016	1.461
THF-ACN 1:1	22.55	0.407	0.56	2.457	0.014	2.443
THF-ACN 1:4	31.52	0.381	0.36	2.625	0.010	2.615
1-octOH	10.3	0.49	0.33	2.041	0.007	2.034
MeOH	32.66	0.36	0.25	2.778	0.007	2.771
DMF	36.7	0.298	0.48	3.356	0.016	3.340
powder	-	0.297	0.52	3.367	0.017	3.349

Table S6. Decay time, $\mathbf{\tau}_{LE}$, and quantum yield, $\mathbf{\Phi}_{LE}$, as well as total, $\mathbf{k_{tot}}$, radiative, $\mathbf{k_r}$, and nonradiative, $\mathbf{k_{nr}}$, rate constant for LE band of the fluorescence spectrum of **C16** in solvents of dielectric constant **ε**.

Solvent	ε	τ_{LE} / ns	Φ_{LE} / %	k_{tot} /ns^{-1}	k_r /ns^{-1}	k_{nr} /ns^{-1}
THF	7.6	0.018	0.027	55.5	0.015	55.5
THF-ACN 1:1	22.55	0.021	0.043	33.3	0.014	33.3
THF-ACN 1:4	31.52	0.081	0.033	12.3	0.004	12.3

Table S7. Deuteration of **C16** effect on fluorescence decay profile parameters in solvents. Amplitudes (**A_1**, **A_2**, **A_3**) and decay times (**τ_1**, **τ_2**, **τ_3**) obtained with deconvolution from fluorescence decay traces of **C16** (proton **P** and deuterated form **D**) recorded at wavelength **λ_o**. Excitation at **λ_{exc}** = 374 nm. Fit parameters: χ^2 and **DW** (Durbin-Watson test) are also provided.

Solvent		λ_o /nm	A_1	A_2	A_3	τ_1 / ns	τ_2 / ns	τ_3 / ns	χ^2	DW
n-hexane	P	540	-0.506	0.494		0.056	0.823		1.078	1.765
	D	540	-0.528	0.472		0.072	0.78		1.128	1.719
THF	P	407	0.959	0.016	0.024	0.018	0.336	0.89	1.209	1.5
	D	407	0.880	0.046	0.074	0.023	0.12	0.77	1.174	1.029
	P	535	-0.485	0.372	0.143	0.053	0.073	0.677	1.278	1.645
	D	535	-0.491	0.372	0.137	0.062	0.083	0.672	1.188	1.693
	P	540	-0.476	0.366	0.158	0.054	0.075	0.679	1.29	1.696
	D	540	-0.487	0.369	0.144	0.062	0.084	0.678	1.17	1.63
THF-ACN 1:1	P	407	0.909	0.086	0.005	0.021	0.37	1.758	1.204	1.384
	D	407	0.863	0.126	0.012	0.035	0.355	1.1	1.36	1.048
	P	530	-0.486	0.348	0.166	0.052	0.079	0.407	1.345	1.368
	D	530	-0.554	0.424	0.022	0.066	0.09	0.412	1.273	1.642
	P	535	-0.484	0.369	0.146	0.056	0.08	0.409	1.323	1.365
	D	535	-0.488	0.376	0.136	0.065	0.087	0.413	1.203	1.633
THF-ACN 1:4	P	407	0.862	0.029		0.081	0.41		1.63	0.534
	D	407	0.719	0.281		0.092	0.368		1.169	1.318
	P	530	-0.494	0.419	0.087245	0.084	0.123	0.381	1.171	1.534
	D	530	-0.495	0.464	0.040846	0.094	0.11	0.378	1.116	1.83

Table S8. Kinetic isotope effect (KIE) of **C16** in solvents calculated for the decay times (τ_1, τ_2, τ_3) taken from Table S7.

Solvent	λ_o /nm	KIE(τ_1)	KIE(τ_2)	KIE(τ_3)
n-hexane	540	1.28		0.95
THF	402	1.17		0.99
	407	1.28		0.87
	535	1.17	1.14	0.99
	540	1.15	1.12	1.00
THF-ACN 1:1	402	1.73		0.97
	407	1.67	0.96	0.63
	530	1.27	1.14	1.01
	535	1.16	1.09	1.01
THF-ACN 1:4	407	1.14	0.90	
	530	1.12	0.89	0.99

Table S9. Fluorescence decay profile parameters of **C16** in solvents forming hydrogen bonds. Amplitudes (**A_1**, **A_2**, **A_3**) and decay times (τ_1, τ_2, τ_3) obtained with deconvolution from fluorescence decay traces recorded at wavelength λ_o. Excitation at λ_{exc} = 374 nm. Fit parameters: χ^2 and **DW** (Durbin-Watson test) are also provided.

Solvent	λ_o /nm	A_1	A_2	A_3	τ_1 / ns	τ_2 / ns	τ_3 / ns	χ^2	DW
1-OctOH	450	0.82	0.16	0.02	0.066	0.329	1.73	1.108	1.753
	550	0.43	0.54	0.024	0.106	0.494	3.05	1.163	1.701
MeOH	437	0.33	0.46	0.21	0.031	0.395	0.827	1.195	1.7
	550	0.21	0.56	0.23	0.046	0.388	1.04	1.178	1.91
DMF	390	0.98	0.02		0.021	0.48		1.383	0.53
	560	-0.49	0.49	0.02	0.03	0.298	1.142	1.136	1.745

Experimental results for Me

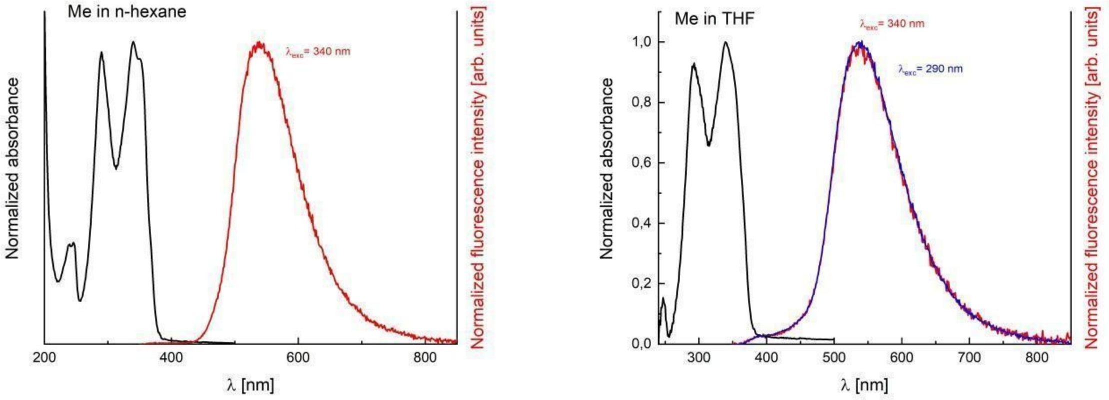

Figure S17. Absorption and fluorescence spectra of **Me** in *n*-hexane and THF at 294 K.

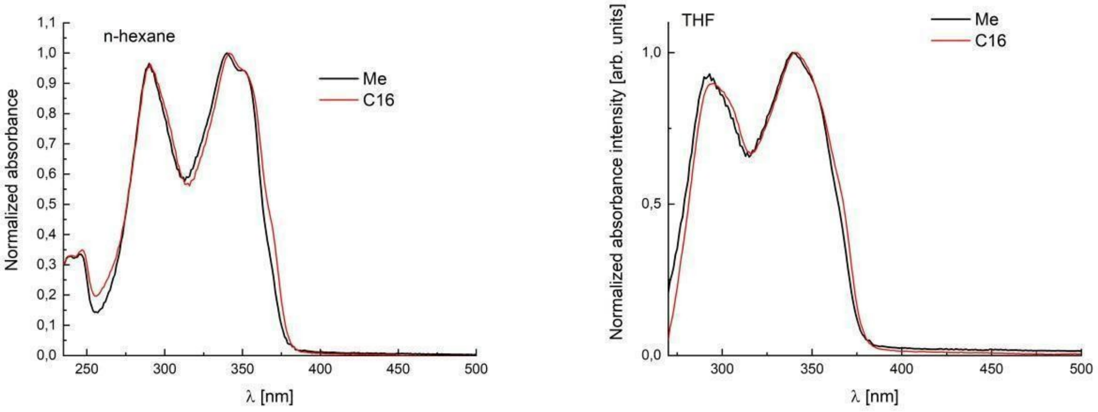

Figure S18. Comparison of absorption spectra of **Me** and **C16** in *n*-hexane and THF at 294 K.

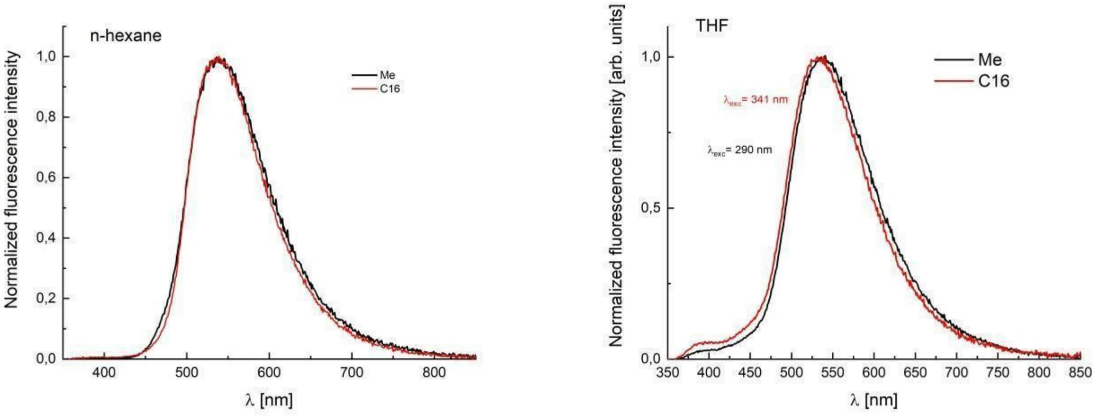

Figure S19. Comparison of fluorescence spectra of **Me** and **C16** in *n*-hexane and THF at 294 K.

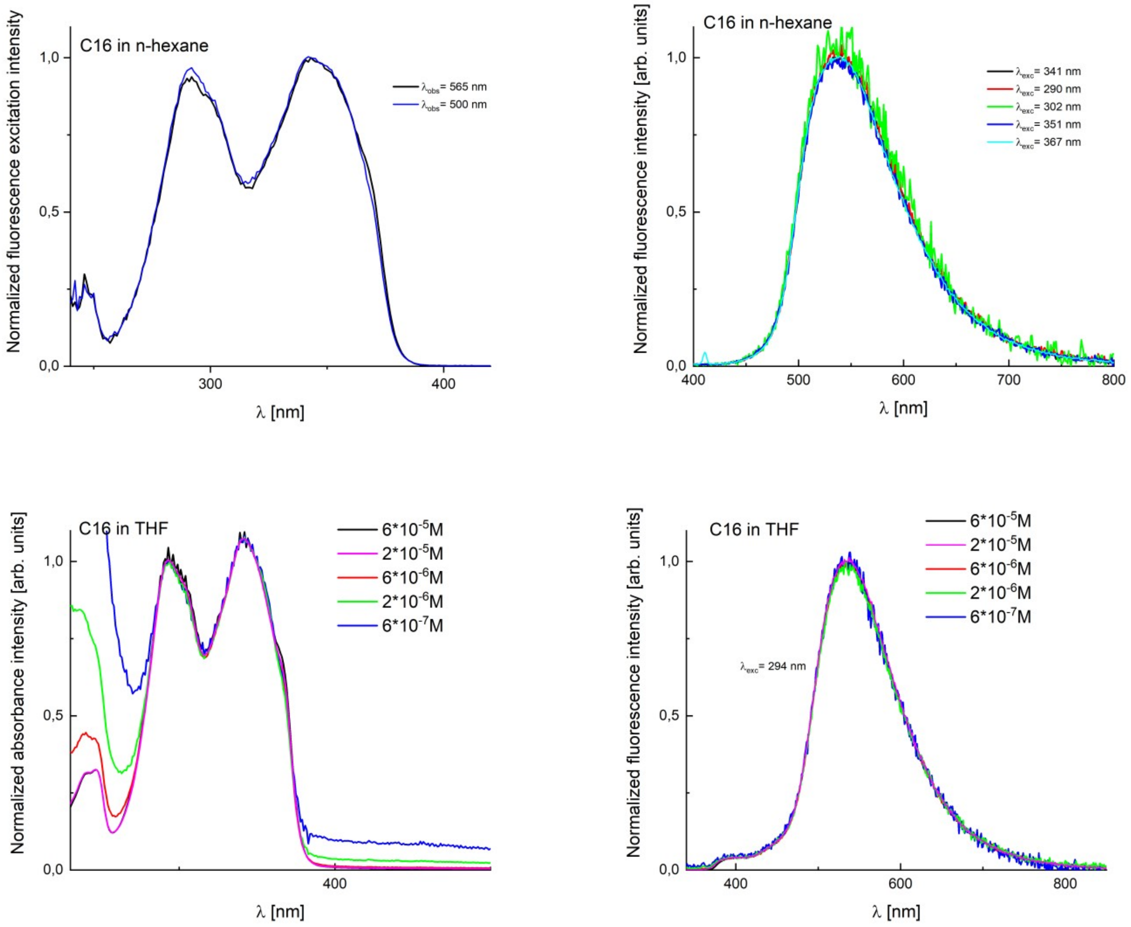

Figure S20. Additional optical spectra of **C16** in non-polar solvents at room temperature. Excitation dependent spectra *n*-hexane (up) and concentration dependent absorption and emission spectra in THF (bottom).

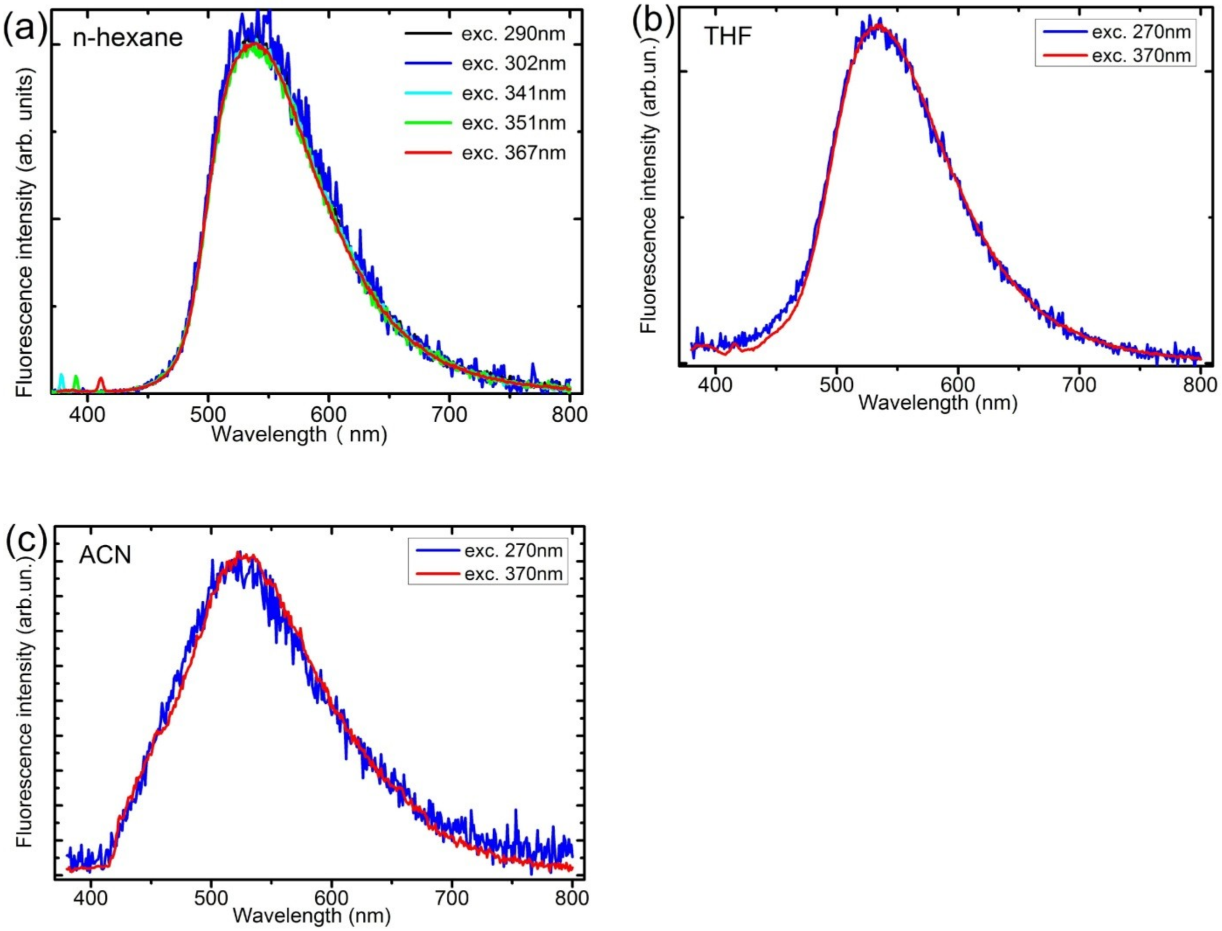

Figure S21. Fluorescence excitation spectra of **C16** recorded at different wavelengths nm – red lines) measured in (a) n-hexane, (b) THF and (c) ACN.

Table S10. Comparison of decay time, **τ**, and quantum yield, **Φ**, as well as radiative, $\mathbf{k_r}$, and nonradiative, $\mathbf{k_{nr}}$, rate constant for ESPT band of the fluorescence spectrum of **C16** and **Me** in selected solvents (*n*-hexane and tetrahydrofuran).

compound	Solvent	$\lambda_{abs\ max}$ / $\lambda_{fl\ max}$ [nm]	Φ [%]	τ / ns	k_r / 10^9s	k_{nr} / 10^9s
**C16**	*n*-hexane	342 / 538	2.6	1.334	0.019	0.730
	THF	341 / 531	1.1	0.59	0.019	1.676
**Me**	*n*-hexane	340 / 539	2.0	1.221	0.016	0.803
	THF	339 / 537	1.0	0.706	0.014	1.402

NMR results for C16

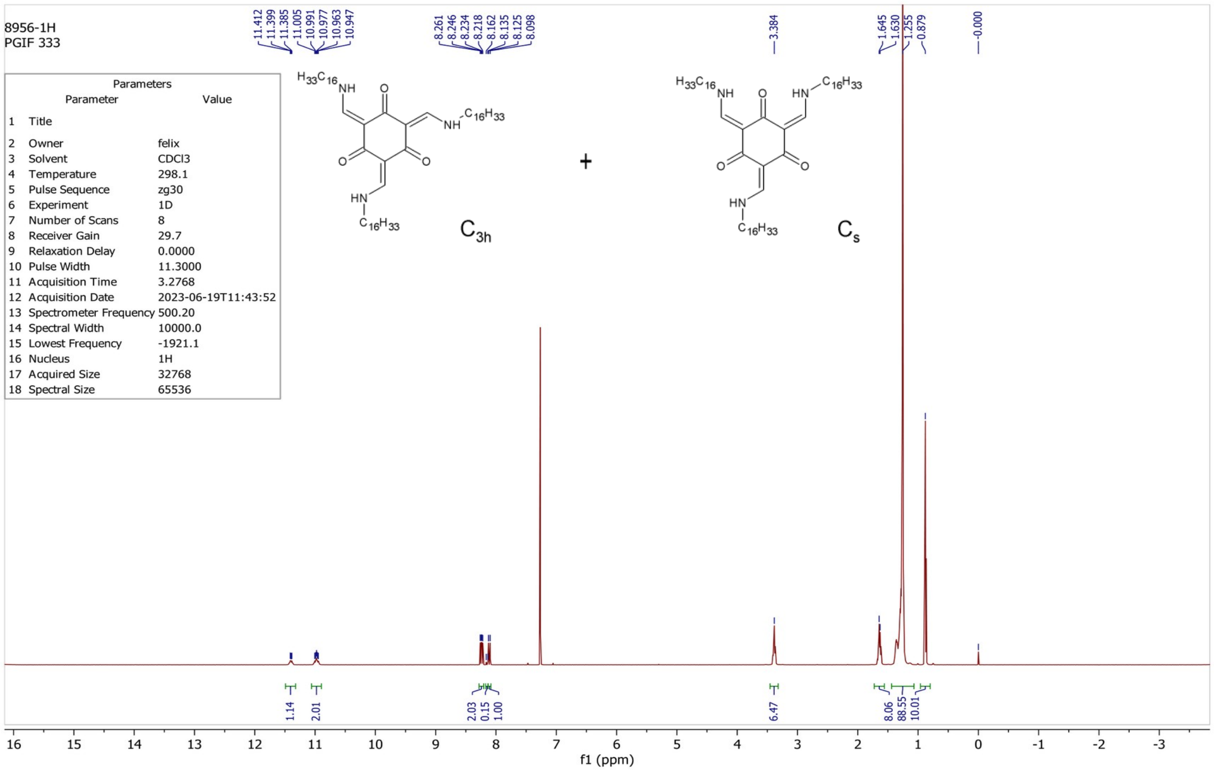

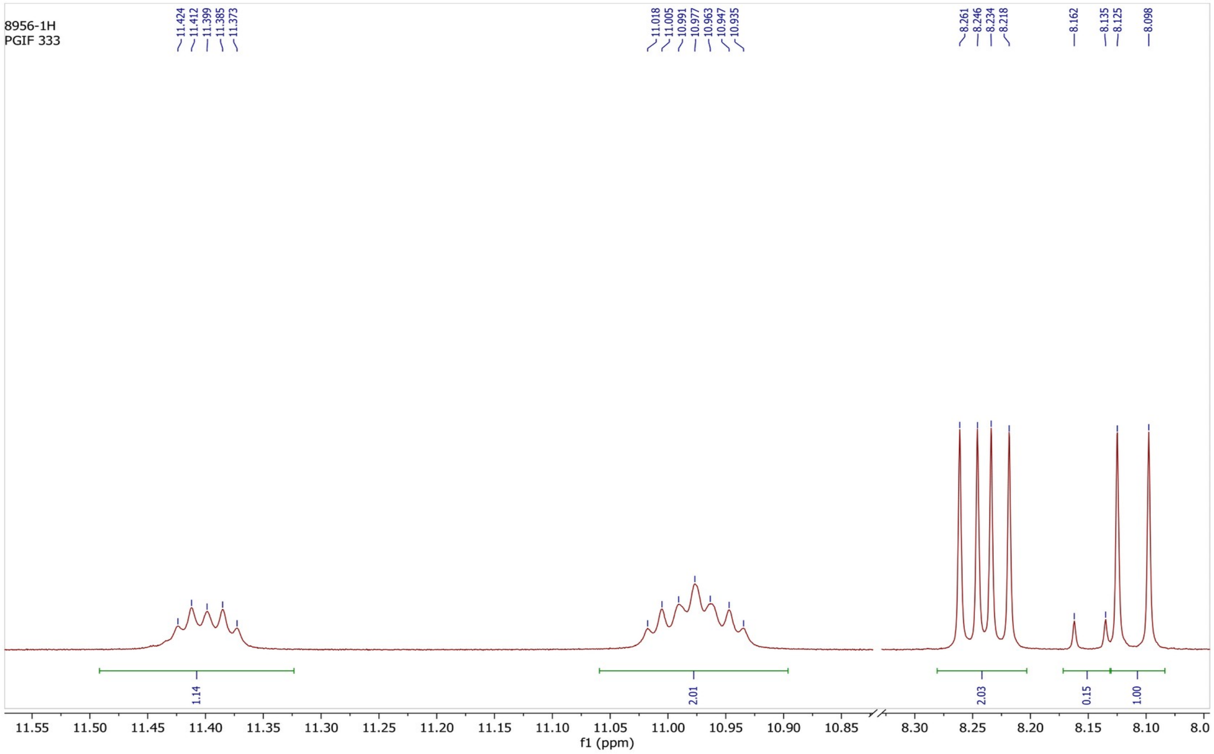

Spectrum S1. ^{1}H NMR spectrum of **C16** (with extended diagnostic region) ($CDCl_3$ +TMS, 298K)

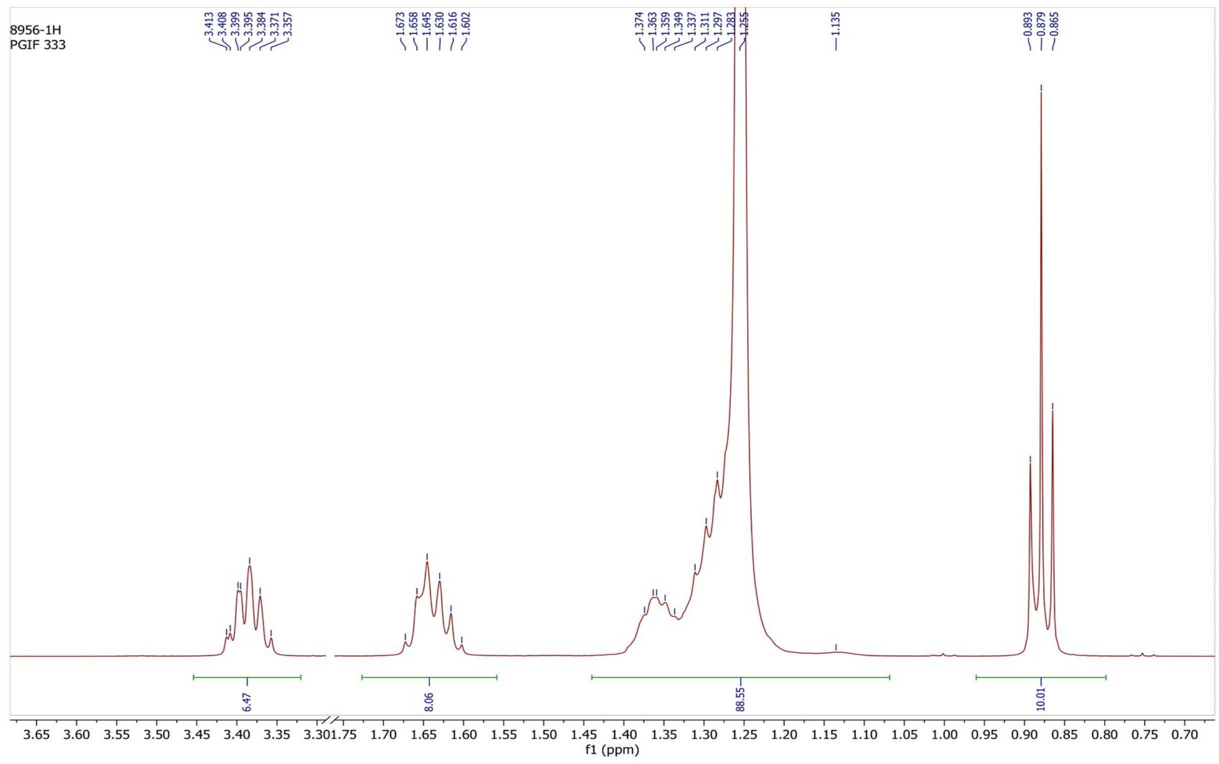

Spectrum S1 (continued). ^{1}H NMR spectrum of **C16** (with extended diagnostic region)

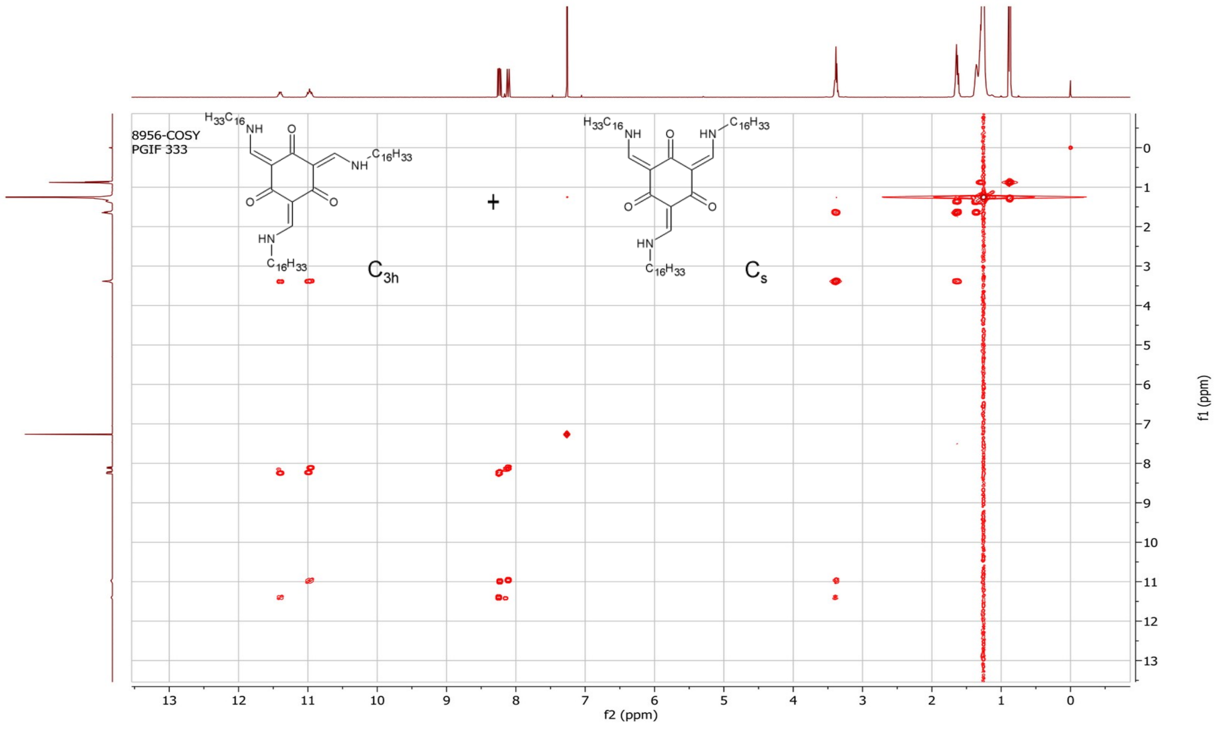

Spectrum S2. ^{1}H-^{1}H COSY NMR spectrum of **C16** (with extended diagnostic region)

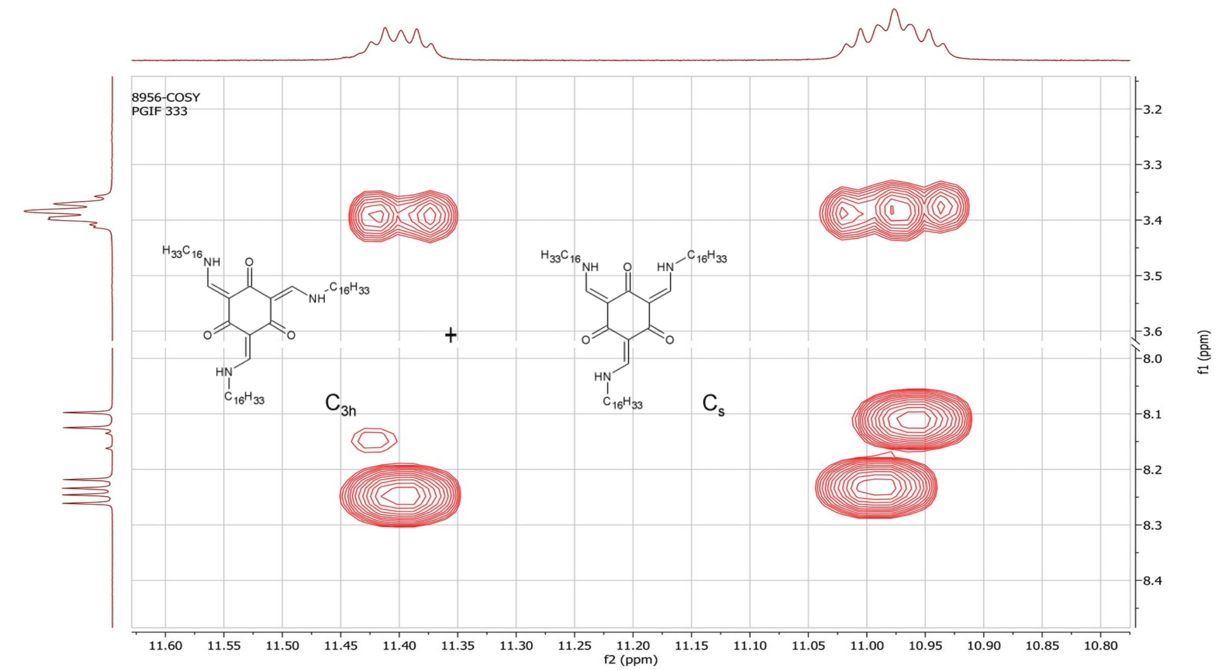

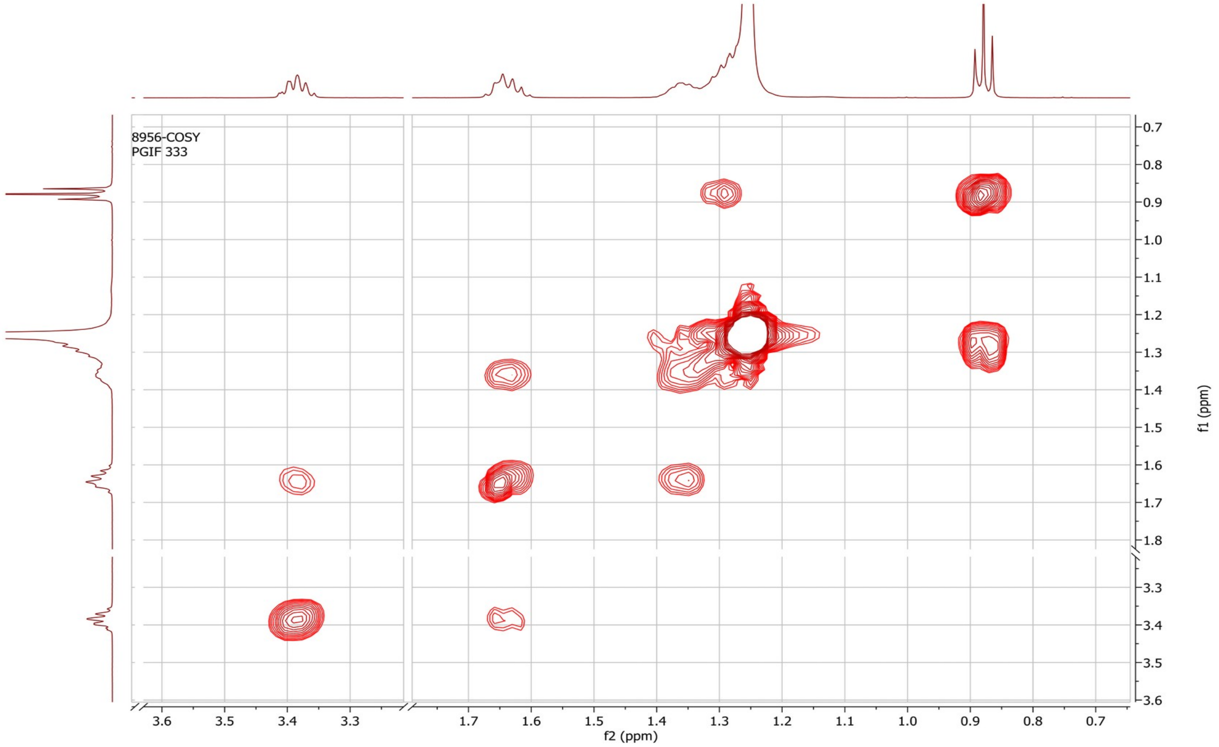

Spectrum S2 (continued). ^{1}H-^{1}H COSY NMR spectrum of **C16** (with extended diagnostic region)

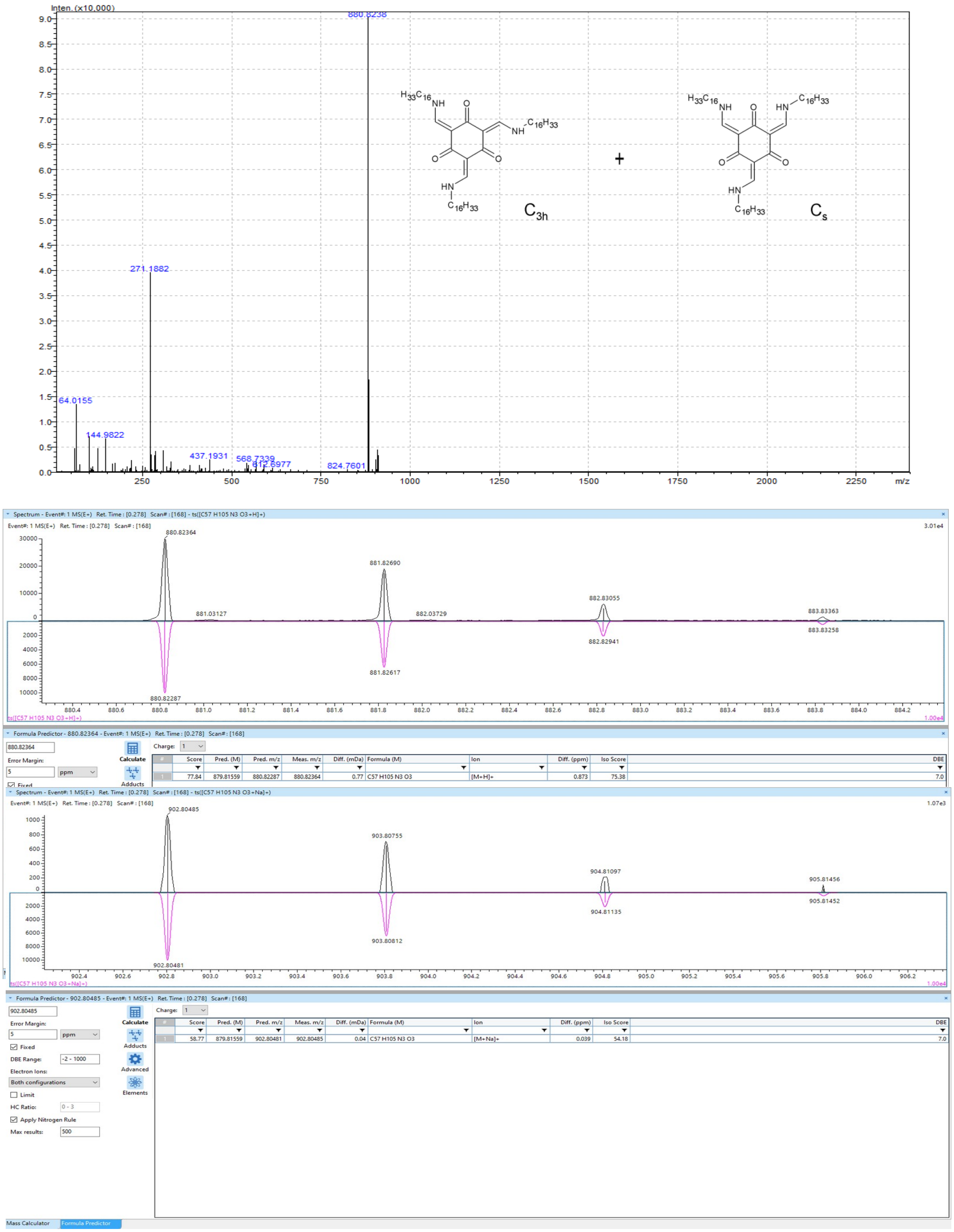

Spectrum S3. HRMS (ESI$^+$) spectrum of **C16** and analytical reports for $[M+H]^+$ and $[M+Na]^+$

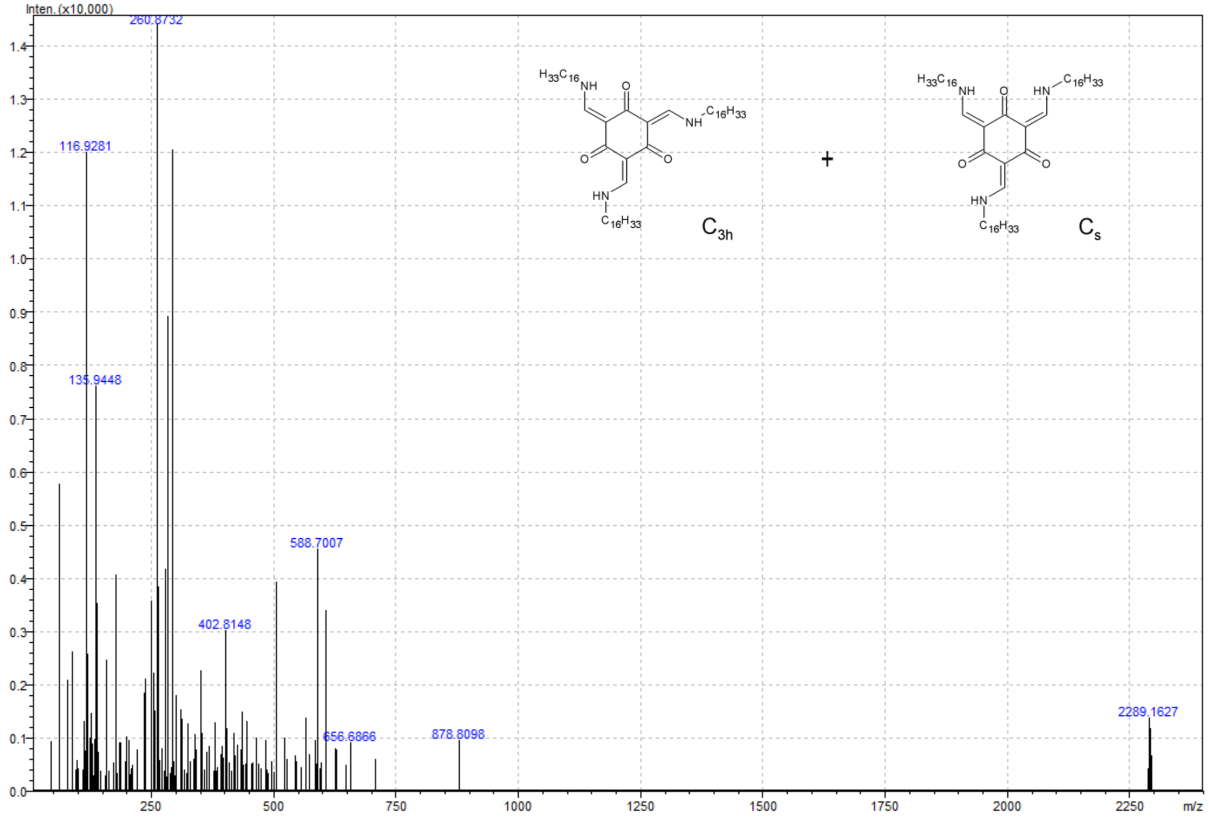

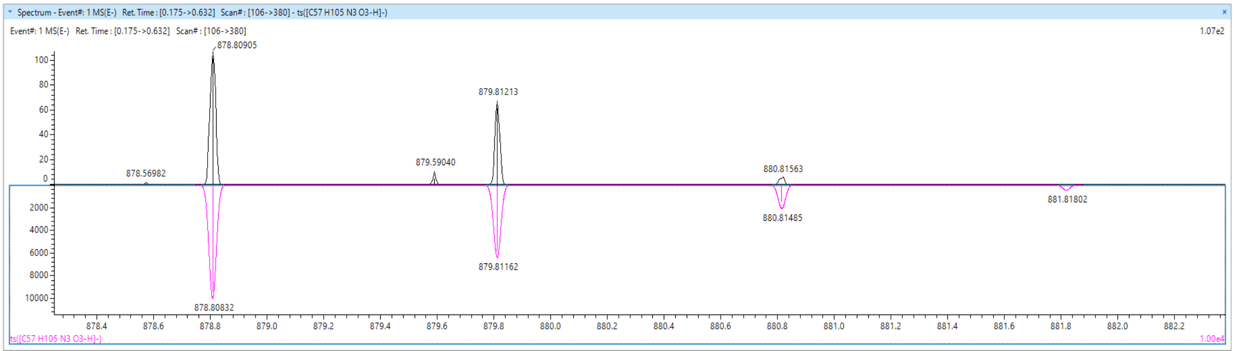

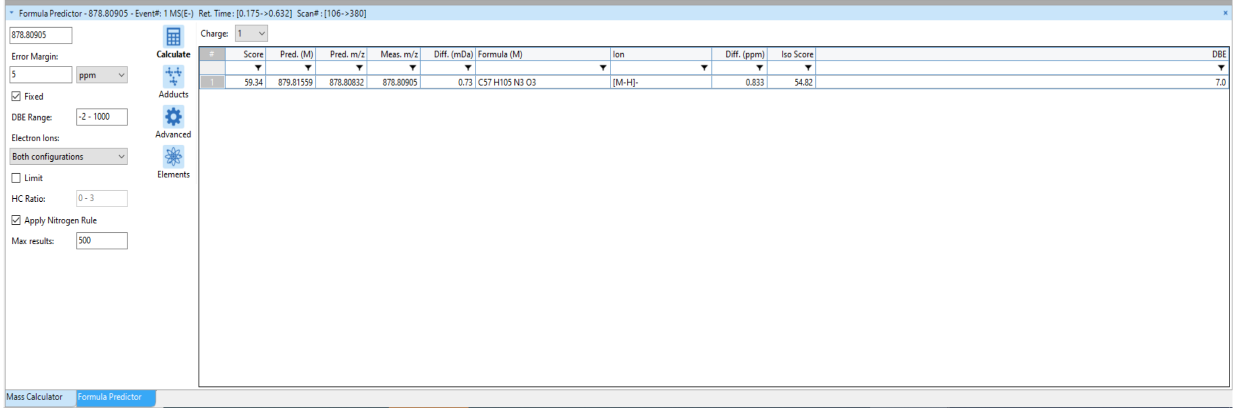

Formula Predictor - 878.80905 - Event#: 1 MS(E-) Ret. Time : [0.175->0.632] Scan# : [106->380]

878.80905
Error Margin: 5 ppm
Fixed
DBE Range: -2 - 1000
Electron Ions: Both configurations
Limit
HC Ratio: 0 - 3
Apply Nitrogen Rule
Max results: 500
Calculate
Adducts
Advanced
Elements
Charge: 1

#	Score	Pred. (M)	Pred. m/z	Meas. m/z	Diff. (mDa)	Formula (M)	Ion	Diff. (ppm)	Iso Score	DBE
1	59.34	879.81559	878.80832	878.80905	0.73	C57 H105 N3 O3	[M-H]-	0.833	54.82	7.0

Mass Calculator
Formula Predictor

Spectrum S4. HRMS (ESI$^-$) spectrum of **C16** and analytical report for [M-H]$^-$

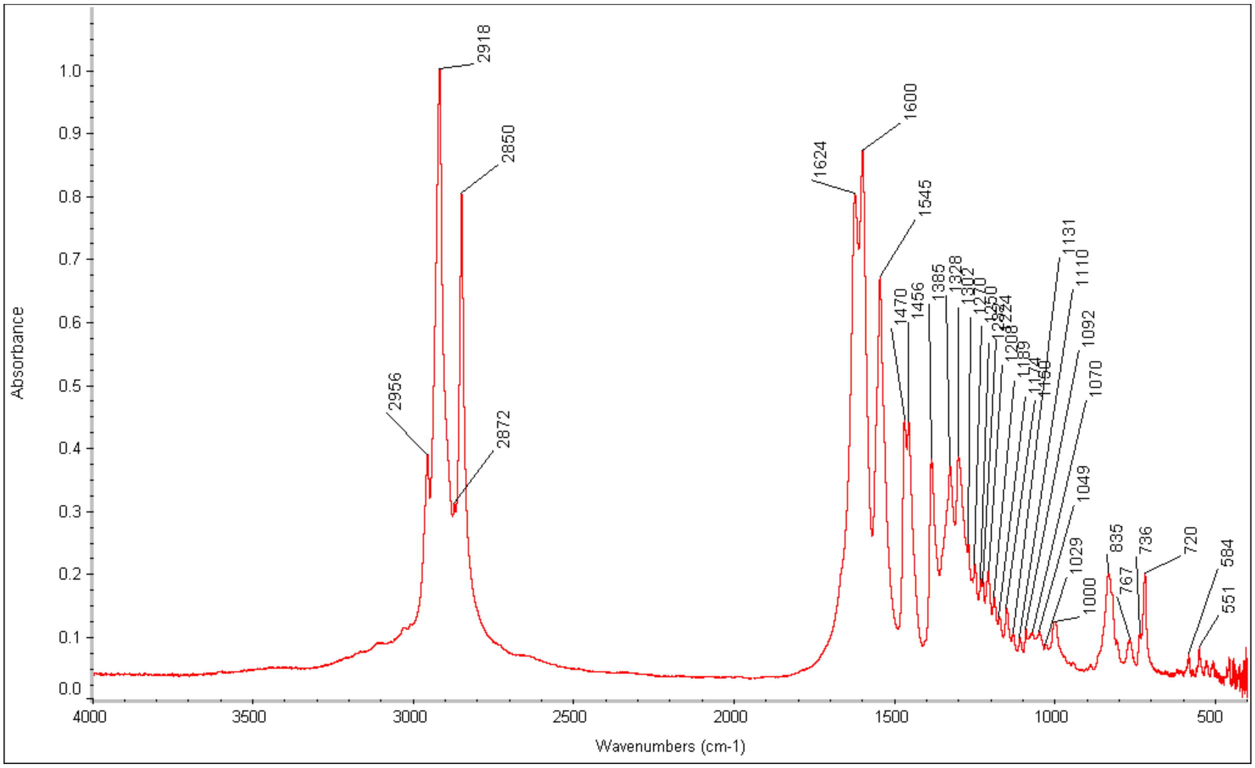

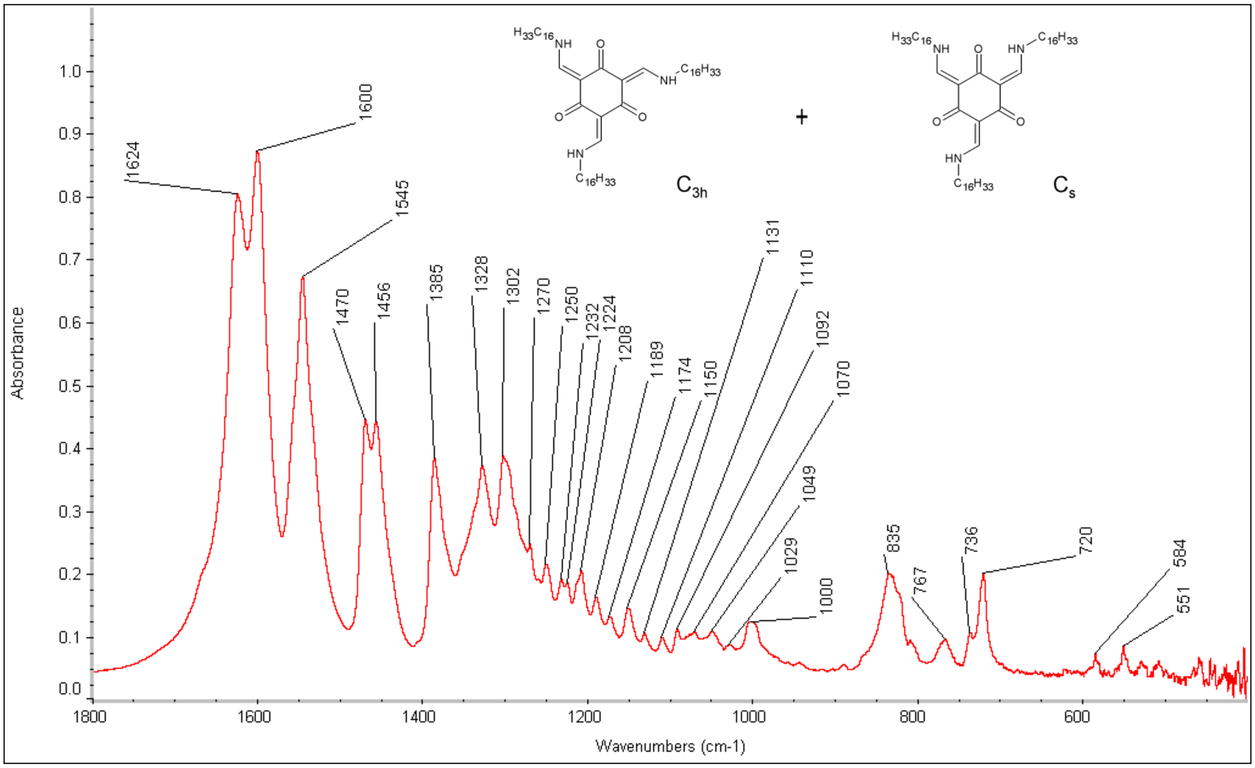

Spectrum S5. IR (KBr) spectrum of **C16**
